# TisB enables antibiotic tolerance in *Salmonella* by preventing prophage induction through ATP depletion

**DOI:** 10.1371/journal.ppat.1013498

**Published:** 2025-09-22

**Authors:** Sebastian Braetz, Niclas Nordholt, Andreas Nerlich, Frank Schreiber, Karsten Tedin, Marcus Fulde

**Affiliations:** 1 Institute of Microbiology and Epizootics, School of Veterinary Medicine, Freie Universität Berlin, Berlin, Germany; 2 Department of Materials and the Environment, Division of Biodeterioration and Reference Organisms (4.1), Federal Institute for Materials Research and Testing (BAM), Berlin, Germany; 3 Veterinary Centre for Resistance Research (TZR), School of Veterinary Medicine, Freie Universität Berlin, Berlin, Germany; Institut Pasteur, FRANCE

## Abstract

Persisters are phenotypically antibiotic-tolerant cells which can survive antibiotic exposure without acquiring antibiotic resistance. A proposed important factor in persistence is low intracellular ATP levels, which are thought to reduce the activity of antibiotic targets. However, previous studies demonstrated that persisters have comparable DNA damage as drug-sensitive bacteria after fluoroquinolone treatment. Furthermore, recent studies reported that endogenous prophages can reduce levels of antibiotic persistence in *Salmonella* after fluoroquinolone treatment. In this study, we examined prophage induction and persister cell survival of a prophage-free variant of *Salmonella* Typhimurium and strains harbouring a deletion of the *tisAB* genes, with *tisB* encoding the toxin from the *tisB*/*istR-1* toxin-antitoxin system, known to reduce the intracellular ATP concentration. Treatment of the prophage-free variant with ciprofloxacin resulted in reduced killing and increased persistence as compared to the wild type. In addition, prophage induction and prophage mediated killing was significantly increased after deletion of *tisAB* following ciprofloxacin treatment. We also demonstrate that the recovery phase following the removal of ciprofloxacin, is crucial for the induction of endogenous prophages. Our results suggest that ATP-dependent prophage activation plays a significant role in DNA damage-mediated killing of bacteria. Low ATP levels can dampen the induction of prophages and increase the fraction of bacterial survivors after ciprofloxacin treatment.

## Author summary

Antibiotic persistence comprises drug-tolerant bacteria that can survive treatment with antibacterial agents, despite lacking classical genetic resistance mechanisms. Therefore, persisters are clinically relevant because they can lead to treatment failures and chronic infections. Additionally, antibiotic persistence facilitates the evolution of resistance through genetic mutations. Persisters are triggered by a lack of nutrients, bacterial toxins, low ATP levels, or other stress responses that shut down bacterial metabolism. However, the involvement of prophages, viruses that integrate into bacterial chromosomes, is less well understood. In this study, we tested a *tisAB* deletion in *Salmonella* Typhimurium and examined persister cell formation following treatment with the DNA-damaging drug ciprofloxacin. TisB is a bacterial toxin that increases the influx of protons across the inner bacterial membrane into the cytosol, causing ATP depletion. We demonstrate that the deletion of *tisAB* increases prophage induction and bacterial killing, leading to a reduced persister cell fraction. The *tisAB* mutant is unable to down regulate its ATP concentration after exposure to ciprofloxacin, which in turn allows for stronger binding of RecA to single-stranded DNA, the activator of both the SOS response and prophage induction.

## Introduction

The phenomenon of antibiotic persistence has been known since the 1940s, first noted when it was observed that cultures of *Staphylococcus aureus* were not completely sterilized after penicillin treatment [1]. Notably, re-cultivation of these persistent bacteria and renewed treatment with penicillin yielded the same killing kinetics, demonstrating that persistence was not heritable, hence no genetically determined resistance mechanisms were responsible for persister cell formation. Renewed interest in persistence emerged 40 years later with the discovery of *hipA*, part of the *hipBA* toxin/antitoxin module, the first genetic locus identified to be involved in persistence following antibiotic treatment [2]. *hipA* encodes a toxin that interferes with translational activity by phosphorylating glutamyl-tRNA synthetase and causing the accumulation of uncharged tRNA leading to reduced translation [2–4]. A number of additional mechanisms and pathways contributing to persistence have since been described, including efflux pumps, protein aggregation, the SOS and oxidative stress responses, toxin-antitoxin (TA) modules, and reduced ATP concentrations [5–11]. In particular, low ATP levels have been reported to play a universal role in persister cell formation [10,11]. A reduced target activity of antibiotics caused by low ATP levels leads to decreased cellular damage following drug treatment, thereby enhancing antibiotic tolerance and contributing to increased bacterial survival [11]. In the same study, the authors further found that, under the conditions tested, ten starvation-induced TA gene modules (encoding interferases with ribosome-dependent and ribosome-independent RNA endoribonuclease activity) previously suggested to be involved in persister cell formation in *E. coli* [12–14], played little or no role in increasing persister populations. Interestingly, studies on the role of the TA modules in *E. coli* were later found to have been confounded by accidental infections of a number of experimental cultures with phage φ80, which led to the misinterpretation of results [12–15]. Re-evaluation of the experiments in prophage-free cultures revealed that the ten RNA interferases were apparently dispensable for persister cell level [15]. However, these observations raised another issue which had been largely overlooked, namely the involvement of endogenous prophages in persister cell formation. In another study, it was demonstrated that prophages have the potential to sensitize bacteria against fluoroquinolones, as demonstrated in the case of *Pasteurella haemolytica* or with an *E. coli* strain lysogenic for bacteriophage λ in combination with danofloxacin [16]. In both cases, treatment with danofloxacin was more efficient in prophage-carrying strains, suggesting that prophages play an important role during drug treatment. Similar results were obtained with *S. aureus*, where tetracycline-mediated inhibition of prophage development through inhibited translation led to increased bacterial survival after ciprofloxacin treatment. [17]. *Salmonella* Typhimurium (ATCC 14028) harbours four SOS response-inducible prophages, designated Gifsy-1, Gifsy-2, Gifsy-3, and ST64B [18–20]. We recently demonstrated that ciprofloxacin mediates a significant part of the killing of *S.* Typhimurium through the induction of endogenous prophages, in particular the expression of the lytic genes of Gifsy-1 [21]. Furthermore, it was also demonstrated that *Salmonella* persisters are killed by prophages during macrophage infection [22].

The toxin TisB, which is part of the TA module *tisB*/*istR-1*, is also activated by the SOS response and is believed to promote persister cell formation by reducing the ATP levels [9,23,24]. TisB is a short peptide, which embeds into the inner bacterial membrane leading to the dissipation of the proton motive force leading to loss of function of the ATP synthase [25,26]. The corresponding antitoxin, IstR1, is a short mRNA that neutralizes the toxicity of TisB by base-pairing with *tisB* mRNA, a typical inhibition mechanism of type I antitoxins that interferes with the translation of the corresponding toxin [27]. Interestingly, the expression of *tisB* also resulted in a decreased transcription and translation, as well as reduced expression of SOS response genes, and induction of bacteriophage λ [23,28]. TisB would therefore appear to be induced by the SOS response and, in turn, provide negative feedback regulation to the SOS response and the induction of prophages.

In this study, we performed experiments with *S*. Typhimurium to investigate the effect of *tisAB* and its associated prophages on persister survival. Our results show that ciprofloxacin-induced *tisAB* expression leads to a reduction in ATP levels, which correlates with reduced prophage induction and increased survival of persister cells.

## Results

### TisB impact on persistence is growth phase-dependent

To investigate the involvement of ATP in regulating persister cell levels in *S*. Typhimurium following exposure to ciprofloxacin, we first verified the correlation between persister cell survival and ATP levels in bacteria sampled at different growth phases, since it is known that actively proliferating bacteria during the exponential phase have higher ATP pools than stationary phase bacteria [10,11,29]. Consistent with previous studies in *E. coli*, samples taken late in the growth phase at higher cell densities showed higher levels of survival (S1A Fig) [30]. Likewise, the increased persistence also inversely correlated with the bacterial ATP levels at the respective growth phases, *i.e.,* the high ATP levels in the exponential phase showed reduced persistence compared to the low ATP levels in stationary phase cultures (S1B Fig). In addition, the membrane potential, which is used to generate ATP [31], also declined with increasing cell densities late in the growth phase, reaching the lowest potential in stationary phase, overnight cultures (S1C Fig).

**Fig 1:**
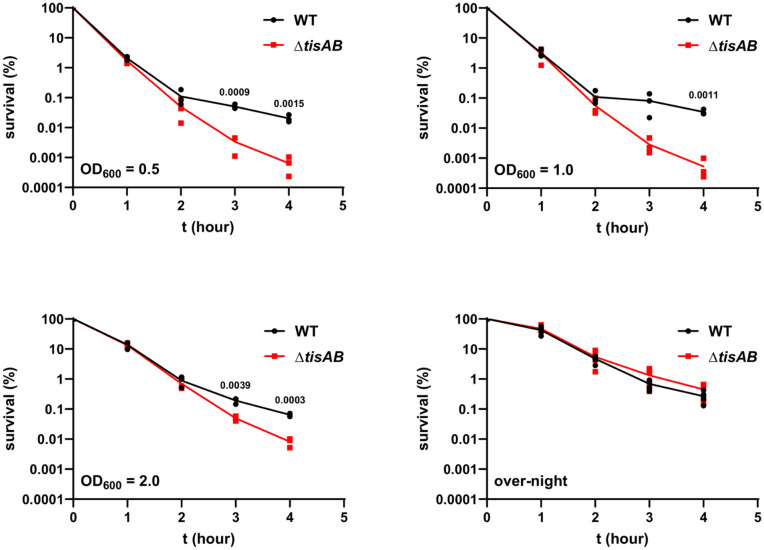
Persister assays at different optical densities. The wild type (8640) and ∆*tisAB* mutant (10752) were incubated in LB medium to the indicated cell density and subsequently treated with four-fold the MIC of ciprofloxacin (1 µg/ml). Significant differences between survival of wild type (black symbols) and ∆*tisAB* (red symbols) at a given timepoint were calculated using an unpaired, two-tailed Student`s t-test. At least three independent experiments were performed.

As TisB is known to reduce the membrane potential, and thus the ATP concentration [23], we therefore compared persister cell levels of *S*. Typhimurium wild type and Δ*tisAB* strains at different points during the growth phase as described above. The persister cell fraction was significantly decreased three and four hours post challenge in the Δ*tisAB* deletion strain compared to the wild type (Fig 1). In contrast, no significant difference was observed in the survival of stationary phase cultures of either the wild type or Δ*tisAB* strains. These results were consistent with previous studies performed with *E. coli* which found that deletion of *tisB* reduced bacterial survival against bactericidal antibiotics in the exponential growth phase and actively growing bacteria, whereas it had no effect on survival of bacteria from overnight cultures [9,32]. As long as the ATP concentration can be reduced, TisB activity has a significant impact on persister cell survival (Fig 1). In addition, we also tested a *tisB* deletion mutant to rule out any potential effect of *tisA* on the persister cell fraction. However, deletion of only *tisB* resulted in the same decreased persister cell fraction as the *tisAB* mutant (S2A Fig). Chromosomal complementation of the *tisAB* mutant also resulted in an indistinguishable persister cell fraction following ciprofloxacin treatment, emphasizing that the observed phenotype is due to *tisB* (S2B Fig).

### Augmented SOS response in the Δ*tisAB* mutant

**Fig 2:**
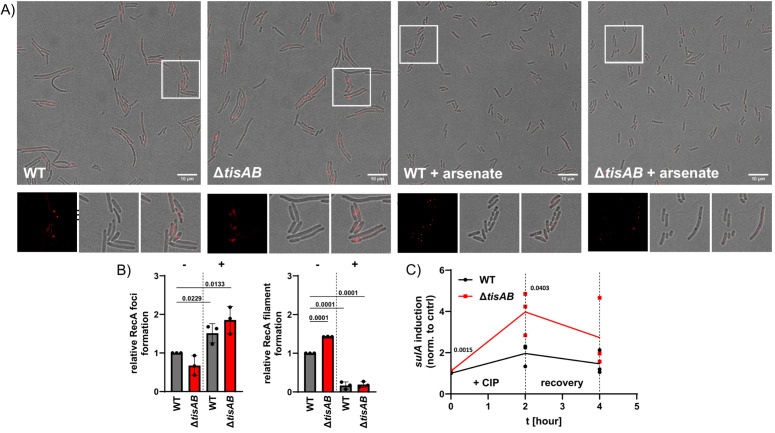
Microscopic images of ciprofloxacin exposed bacteria with recA-mScarlet-I fusion and *sulA* expression. A) Representative images of bacteria exposed to ciprofloxacin. The strains (SB448 = wild type and SB451 = Δ*tisAB*) were incubated to mid-log phase (OD_600_ = 0.5) and subsequently added onto LB soft agarose pads containing 1 µg/ml ciprofloxacin, followed by further incubation for two hours at 37°C before imaging. Where indicated, the bacteria were pre-treated for 20 minutes with 0.5 mM arsenate to reduce ATP concentration. B) RecA foci and filamentation, as shown in A), were blindly quantified and normalized to the wild type (SB448). - = no arsenate; + = 0.5 mM arsenate. C) Determination of *sulA* expression to quantify the induction of the SOS response. The wild type (8640) and *tisAB* mutant (10752) were treated for two hours with 1 µg/ml ciprofloxacin, followed by a recovery phase without antibiotics. Experiments were performed independently three times, and significance was calculated using the unpaired Student’s t-test.

The gene *tisAB* is regulated by the SOS response, and its overexpression in *E. coli* has been shown to reduce SOS activation, suggesting a regulatory effect on the bacterial DNA repair system [28]. Therefore, to determine the possible effects of TisB on the SOS response in *S*. Typhimurium, we constructed mutants in which *recA*, the inducer of the SOS response, was fused to the fluorescent protein mScarlet-I in the chromosome for microscopic studies. A similar RecA construct was generated in *E. coli*, in which RecA was fused to YFP (yellow fluorescent protein) and retained its full function [33]. The corresponding wild type and *tisAB* mutant were incubated until mid-log phase, diluted, and pipetted onto LB soft agar pads containing ciprofloxacin. After an additional two hours of incubation at 37°C, we examined the activation of RecA-mScarlet under the microscope (Fig 2A). Interestingly, the mScarlet-I signal appeared predominantly as filamentous structures throughout the cytosol, particularly in the *tisAB* mutant, resembling RecA-YFP filamentation in *E. coli* [33]. In addition to apparent RecA filamentation, we also observed red foci, suggesting protein aggregation (Fig 2A). We quantified both observations (see materials and methods) and determined significantly increased RecA filamentation in the *tisAB* mutant, whereas RecA foci formation was non-significantly reduced (Fig 2B). As low ATP levels can lead to protein aggregation within the cytosol [34], we also pre-treated the wild type and the *tisAB* mutant with 0.5 mM arsenate to artificially reduce ATP concentration [10,11,29,35]. Pre-treatment with arsenate prior to ciprofloxacin treatment resulted in shorter bacterial cells, and the RecA-mScarlet-I fusion protein formed red foci within the cytosol, as expected under low ATP conditions (Fig 2A). Furthermore, arsenate decreased RecA filamentation in both the wild type and the *tisAB* mutant (Fig 2B). These results indicate that activation of *tisAB* upon treatment with ciprofloxacin leads to low ATP levels and RecA aggregation and reduced RecA binding to the DNA, i. e., RecA filamentation on the DNA. This observation is further supported by *tisB* overexpression experiments performed by Leinberger *et al*. (2024), in which artificial induction of *tisB* led to protein aggregation [36]. We therefore sought to investigate whether *tisAB*, which is expressed as part of the SOS regulon, can influence the strength of SOS induction after activation of RecA by DNA damage. To determine the intensity of the SOS response, we measured the transcriptional upregulation of the RecA-dependent SOS gene *sulA* [37]. We treated the wild type and *tisAB* mutant with ciprofloxacin for two hours, followed by RNA extraction. In addition, we also extracted the RNA during the recovery phase, when the antibiotic was removed and the bacteria recovered from the drug treatment. This phase is crucial for persister cell survival, as bacteria activate their SOS response system during this time [38]. *sulA* expression was higher in the *tisAB* mutant after two hours of ciprofloxacin treatment, indicating that *tisAB* has an inhibitory and significant effect on the induction of the SOS response (Fig 2C). *sulA* expression was also slightly but significantly upregulated (approximately by 10%) during exponential growth in the *tisAB* mutant before the addition of ciprofloxacin (t0 in the figure), indicating that *tisAB* also regulates the SOS response under normal growth conditions. Together, these findings suggest that *tisAB* acts as a negative regulator of the SOS response, both under normal conditions and during antibiotic-induced stress.

**Fig 3:**
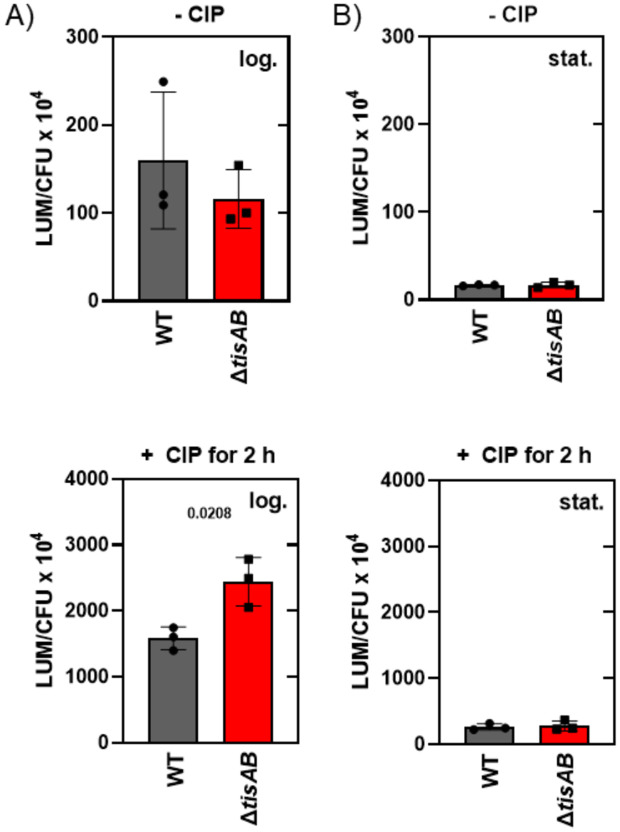
ATP levels before and after treatment with ciprofloxacin. A) Exponentially growing (OD_600_ = 0.5), and B) stationary phase cultures (overnight) of either the wild type (8640, grey bars) or ∆*tisAB* mutant (10752 red bars) were treated for two hours with 1 µg/ml ciprofloxacin before ATP determination. The generated luminescence signal was normalized to the colony-forming units (CFU) before the addition of ciprofloxacin, in order to account for dying bacteria that also contribute to an ATP-dependent signal. In addition, the ATP levels of untreated bacteria were also measured. Three independent experiments were conducted, and significance was calculated using the unpaired Student’s t-test.

It has been reported that upon DNA damage, ATP concentration rapidly increases, followed by a RecA-dependent ATP decrease, indicating that RecA activates a mechanism to reduce intracellular ATP levels [39–41]. To determine how TisB would affect the ATP pools in response to ciprofloxacin, we determined ATP levels in wild type and Δ*tisAB* strains following a two hour treatment with ciprofloxacin. There was no significant difference between the untreated wild type and Δ*tisAB* strains during exponential growth (Fig 3A). In contrast, the Δ*tisAB* mutant showed approximately 1.5-fold more ATP than the wild type after challenge with ciprofloxacin (Fig 3A). These results are in agreement with previous studies where over-expression of TisB reduced the ATP concentration and inhibited the expression of SOS response genes [23,28]. The same experiments performed with stationary phase cultures showed no significant differences, consistent with the low ATP levels observed in stationary phase (Fig 3B). These results suggest an ATP-dependent and TisB-mediated regulation of the intensity of the SOS response during the exponential phase. We also tested the complemented *tisAB* strain as well as the *tisB* single mutant. The complemented strain was able to regulate its ATP levels after ciprofloxacin exposure, whereas the *tisB* single mutant showed a nearly twofold increase in ATP levels, indicating that *tisB* is a key factor for ATP regulation during ciprofloxacin treatment (S3A Fig).

### Persister cell survival of prophage-free strains upon ciprofloxacin exposure

**Fig 4:**
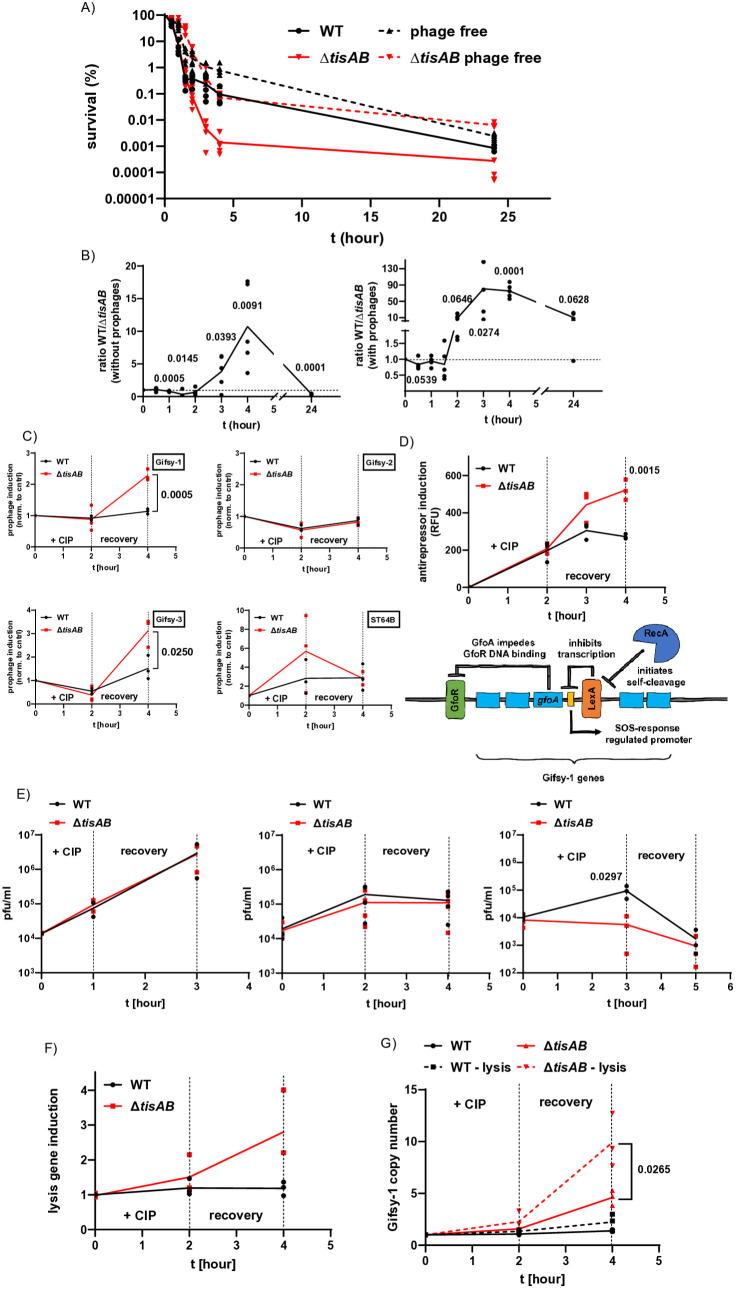
Increased persister formation in a prophage-free *tisAB* mutant and prophage induction. A) Exponentially growing (OD_600_ = 0.5) bacteria (prophage-positive wild type = 8640, prophage-positive Δ*tisAB* = 10752, prophage-free wild type = 11126, prophage-free Δ*tisAB* = 11588) were treated with four-fold the MIC of ciprofloxacin in fresh LB medium and survival was determined at the indicated time points. B) Calculation of the fold change between the prophage-carrying wild type and the corresponding ∆*tisAB* mutant and prophage-free variants (calculated using the survival rates of panel A)). C) Transcriptional activation of the four indicated prophages in *S*. Typhimurium. The strains were treated for two hours with 1 µg/ml ciprofloxacin, followed by a recovery phase in LB. The results were normalized to the untreated wild type (8640). D) The antirepressor of Gifsy-1 (*gfoA*) was replaced with mCherry to quantify induction following ciprofloxacin treatment (wild type = SB287, Δ*tisAB* = SB288). The bacteria were treated as indicated in the figure, washed, and resuspended in PBS for FACS analysis. At least 500,000 events per experiment were measured. The average mCherry signal was calculated and plotted against the time points indicated in the figure. The illustration below depicts how Gifsy-1 is activated. After RecA-mediated cleavage of LexA, the expression of the antirepressor gene *gfoA* is induced. GfoA then binds to the Gifsy-1 repressor (GfoR), causing its dissociation from the DNA and leading to the induction of the remaining prophage genes. E) Plaque assays were conducted using MS1487 and its corresponding *tisAB* mutant (SB521) following ciprofloxacin treatment, which included an additional recovery phase. F) RT-qPCR results to assess the expression of the *Rz* lysis gene, homologous to lambda’s *Rz* gene. G) Determination of Gifsy-1 copy number by comparing strains with and without lysis genes to assess Gifsy-1 DNA accumulation (strains 11326/SB149 vs. SB522/SB523). Here, “− lysis” denotes the deletion of *SRRz*, responsible for lysis after prophage induction. At least three independent experiments were conducted for each assay. Significant differences were calculated using an unpaired, two-tailed Student`s t-test.

Deletion of *tisAB* resulted in increased killing after a challenge with ciprofloxacin (Fig 1), and a deregulation of the SOS response (Fig 2C). The SOS response is involved in the activation of endogenous prophages, which in turn can contribute to the bacterial killing by bactericidal antibiotics or during macrophage infection [21,22]. We therefore investigated whether activation of endogenous prophages might underlie the observed increased killing in Δ*tisAB* mutants. The wild type ATCC 14028s used in this study harbours Gifsy-1, Gifsy-2, Gifsy-3, and ST64B, which are inducible and activated by the SOS response [19,20,42–44]. To investigate a possible role for prophage induction in persister cell survival due to activation of the SOS response in the Δ*tisAB* background, we introduced the Δ*tisAB* deletion into strains harbouring deletions of the four endogenous prophages, Gifsy-1, Gifsy-2, Gifsy-3, and ST64B, and conducted persister assays in exponentially growing cultures, comparing the original wild type strain (prophage positive) and Δ*tisAB* (prophage positive) mutant to prophage-free counterparts. The initial killing kinetics upon ciprofloxacin challenge were the same for both the wild type and the Δ*tisAB* mutant during the first 2 hours of treatment (prophage positive strains). However, the Δ*tisAB* mutant showed an approximately 100-fold increased killing after four hours of treatment (Fig 4A). In contrast, the same assays performed with the prophage-free variants showed a delayed initial killing for the Δ*tisAB* strain compared to the prophage-free wild type strain (Fig 4A and 4B). Deletion of Δ*tisAB* in the prophage-carrying wild type reduced persister cell fraction 80-, 75-, and 10-fold at three, four, and 24 hours post-challenge, respectively (Fig 4B). In contrast, persister cell levels were only compromised four-fold and ten-fold at three and four hours after treatment with ciprofloxacin in the prophage-free background (Fig 4B), suggesting that TisB has an inhibitory effect on prophages and, consequently, influences the overall persistence of bacterial cells. Interestingly, survival of the prophage-free Δ*tisAB* mutant was two-fold increased compared to the corresponding wild type 24 hours post-challenge (Fig 4A and 4B). With the prior calculated fold change differences, we also calculated the contribution of prophage-mediated reduction of persister levels after deletion of *tisAB*. For example, three hours post-challenge we observed 80-fold less *tisAB* persisters when prophages were still integrated in the chromosome, compared to a four-fold reduction of persisters when prophages were deleted in the *tisAB* mutant (Fig 4B). These results indicated that 95% of the difference in the fraction of persister cells between the wild type and the Δ*tisAB* mutant was attributable to the induction of prophages and 5% due to other mechanisms. After four hours of treatment, prophage induction was responsible for 87% of the difference between wild type and mutant. This suggests that the low ATP levels restricts the consequences of prophage induction. To exclude any effect on *tisAB* expression after the deletion of prophages, we determined *tisB* expression in the prophage-carrying wild type and the respective prophage-free variant following ciprofloxacin treatment. In both strains, *tisB* expression was strongly induced, approximately 200-fold compared to the untreated control, indicating that *tisB* expression is not negatively affected by the deletion of prophages (S3C Fig). Furthermore, the involvement of ATP in prophage-mediated killing is further supported by persister assays performed with bacteria from over-night cultures where ATP levels are low (S4A Fig). These experiments showed no difference between the wild type (with prophages) and the corresponding prophage-free variant (S4A Fig). These results are also in agreement with results shown in Fig 1, where no differences were observed between the wild type and Δ*tisAB* strains using bacteria from overnight cultures. Furthermore, the prophage-free variant of *tisAB* is unable to control its ATP levels when treated with ciprofloxacin. Similar to the prophage-positive variant, it also exhibits a nearly twofold increase in ATP concentration (S3B Fig). However, the persister cell fraction of the prophage-free *tisAB* mutant is increased despite high ATP levels (Fig 4A).

To verify the effects of TisB on induction of the prophage, we performed quantitative real-time PCR to determine the transcriptional activation of Gifsy-1, Gifsy-2, Gifsy-3, and ST64B by targeting the genes STM2605, STM1048, SspH1E3, and sb41 which are encoded within the respective prophages. As previously performed for *sulA* expression, we treated the bacteria with ciprofloxacin for two hours, followed by RNA extraction to assess prophage induction. Additionally, we washed the bacteria and incubated them in LB without antibiotics (recovery phase). For Gifsy-1, Gifsy-2, and Gifsy-3, we observed no induction after two hours of treatment. ST64B was upregulated in both the wild type and the *tisAB* mutant compared to the untreated control (Fig 4C). During the recovery phase, ST64B activation was down regulated. In contrast, Gifsy-1 was up regulated three-fold in the *tisAB* mutant but only weakly induced in the wild type. Gifsy-2 was not activated in either strain, whereas Gifsy-3 showed stronger up regulation in the *tisAB* mutant. In a previous study, we found that Gifsy-1 significantly affects persister levels following ciprofloxacin treatment, whereas the other three prophages appeared to play no role [21] (the lack of impact of ST64B induction is discussed in the Discussion section). Deletion of Gifsy-3 had no effect on persister survival as long as Gifsy-1 remained integrated in the *Salmonella* chromosome [21]. To further investigate potential prophage induction in the *tisAB* mutant, we replaced the *gfoA* gene of Gifsy-1, encoding a RecA-dependent antirepressor [43], with mCherry to monitor promoter activity. Note that this reporter reflects only *gfoA* promoter activity and not the expression of other prophage genes. We treated the bacteria as before and analyzed the mCherry signal using FACS analysis with at least 500,000 events. FACS analysis confirmed a stronger induction of the prophage antirepressor in the *tisAB* mutant, with the most pronounced differences observed during the recovery phase (Fig 4D) consistent with the transcriptional upregulation of Gifsy-1 (Fig 4C). To test whether the augmented translational activity is a general effect of increased ATP levels in the *tisAB* mutant, we repeated the experiments in strains harbouring a chromosomal ribosomal protein gene promoter P*rpsM*::*gfp* reporter. Upon treatment with ciprofloxacin, the GFP signal increased similarly to mCherry. However, during the recovery phase, GFP expression was down regulated in both strains (S5 Fig), in contrast to mCherry expression in the *tisAB* mutant. This suggests that the increased mCherry induction is ATP-dependent via RecA, and that elevated ATP levels alone do not necessarily lead to a general increase in translation in the mutant during the recovery phase. After demonstrating increased induction of Gifsy-1 in the *tisAB* mutant, we sought to perform plaque assays. Unfortunately, our strains did not form visible plaques following drug treatment. A similar issue was reported by Figueroa-Bossi and Bossi, where Gifsy phages formed only small plaques, making quantification difficult [44]. The cause of the inefficient plaque formation remains unclear. We therefore performed these assays in a *Salmonella* Typhimurium ATCC 14028 strain (MS1487) which harbours Gifsy-1 and which has been previously reported as capable of forming visible plaques [22]. We introduced our *tisAB* deletion into MS1487 and conducted plaque assays. Bacteria were treated with ciprofloxacin for one, two, or three hours, followed by an additional two-hour recovery phase in LB medium. Treatment for one hour followed by recovery resulted in increased plaque formation, which further increased during the recovery phase, reflecting the heightened drug sensitivity of bacteria at the early stage of treatment (Fig 4E). After two hours of treatment, plaque formation reached a plateau, resembling the plateau seen in persister cell survival. Following three hours of ciprofloxacin treatment, plaque formation of the wild type decreased during the recovery phase. These results suggest that plaque formation is dependent on drug sensitivity. Persister cells, which form after three hours of ciprofloxacin treatment, down regulate prophage induction, whereas drug-sensitive bacteria upregulate them (compare one hour to three hours of treatment). Thus, our data support the idea that persister cells produce fewer plaques than non-persister cells, as evidenced by the reduced phage release observed in populations enriched for persisters. It also indicates that released phages do not infect viable bacteria as long as they carry the same prophage, thereby preventing superinfection, consistent with previously reported observations [44]. However, to our surprise, the *tisAB* mutant produced significantly fewer plaques after three hours of treatment. To explain this contradictory observation, we therefore tested the expression of the lysis gene *Rz*, which, together with *S* and *R*, is responsible for the lysis of the bacterial host by Gifsy-1 upon induction [21]. Fig 4F shows that *Rz* expression is at least twofold higher in the *tisAB* mutant. This could explain the lack of observable differences in the plaque assays, as overexpression of lysis genes may lead to premature cell death, which in turn could impair proper phage assembly [45,46]. To support increased lysis gene-mediated killing in the *tisAB* mutant, we used an alternative approach. We treated strains carrying Gifsy-1 either with or without lysis genes (*SRRz*) and compared Gifsy-1 DNA accumulation via qPCR. After two hours of ciprofloxacin treatment, we observed a sharp increase in Gifsy-1 DNA in the *tisAB* mutant, whereas the wild type strain showed only a moderate increase (Fig 4G). We also calculated the DNA accumulation difference between strains with and without lysis genes. In the *tisAB* mutant, deletion of *SRRz* increased Gifsy-1 DNA accumulation by 2.2-fold, while in the wild type the increase was 1.6-fold, suggesting that loss of *tisAB* may enhance prophage-mediated lysis. This is consistent with the observed enhanced replication of Gifsy-1 and derepression of its lysis genes in Δ*tisAB*, as well as increased survival following prophage deletion after ciprofloxacin treatment.

### Reduced ATP levels increase survival upon UV irradiation

**Fig 5:**
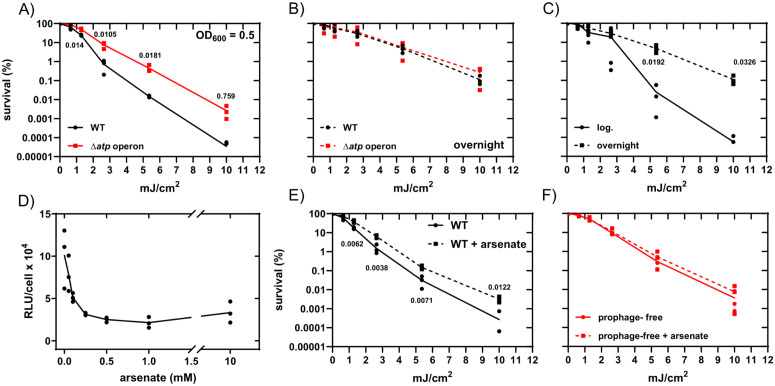
UV survival assays and ATP determination. A) Survival of the wild type (8640) and ∆*atp* mutant (9200) after UV irradiation, which were either incubated to the mid. log phase (OD_600_ = 0.5) or B) overnight. C) Survival of the wild type incubated either to the mid. log phase or stationary phase. D) ATP levels of *S*. Typhimurium incubated to OD_600_ = 0.5 in LB after addition of arsenate at different concentrations. E) UV survival of the prophage-positive strain and F) the prophage-free variant (11126) after incubation to the mid. log. phase either with or without 0.5 mM arsenate. Significant differences were calculated using the unpaired student`s t-test (two tailed). Three independent experiments were performed.

Previous studies have shown that DNA damage and activation of the SOS response in *E. coli* resulted in a sharp, transient increase in the ATP concentrations [39,41]. An *S*. Typhimurium *atp* operon deletion mutant (Δ*atp*) contains approximately 50% of the ATP levels compared to the wild type strain [29,47]. Furthermore, the membrane potential is approximately 12% higher in the *S*. Typhimurium Δ*atp* strain relative to the wild type (S4B Fig), consistent with prior observations with *E. coli atp* mutants [48]. To determine whether a high membrane potential or high/low ATP concentration is important for survival upon DNA damage, we incubated the wild type and Δ*atp* strains to the mid-log. growth phase and subjected the bacteria to UV irradiation. Exposure to UV resulted in ten- to 100-fold increased survival of the ∆*atp* operon mutant compared to the wild type, indicating survival is highly ATP dependent after DNA damage and irrespective of the membrane potential (Fig 5A). In support of this, there was no difference in UV tolerance between the ∆*atp* operon mutant and wild type in overnight cultures, when ATP levels are low (Fig 5B) [29]. Likewise, exponential phase-cultures were more susceptible to UV (Fig 5C).

In order to verify our observations regarding ATP-dependent survival, we also used arsenate to artificially reduce the ATP concentration in exponentially growing bacteria, as has been done in previous studies [10,11,29]. As shown in Fig 5D, arsenate concentrations of 0.5 to 10 mM added to cultures of the wild type strain resulted in similar, strong reductions in ATP levels. To determine the effects of ATP depletion on UV sensitivity and the induction of prophages, we treated exponential growing prophage-free bacteria with 0.5 mM arsenate for 30 min, followed by removal of samples for UV irradiation and plating to LB plates to determine the survival. Pre-treatment with arsenate of the prophage-carrying wild type resulted in increased tolerance against UV-induced DNA damage (Fig 5E). In contrast, ATP depletion had no effect on survival in the prophage-free strain background (Fig 5F), consistent with ATP-dependent activation of prophages upon DNA damage. To exclude the possibility that the reduced ATP concentration influenced growth recovery and to reinforce the idea that survival was due to the inhibition of prophage activation, we also measured the duration of the lag phase before and after UV irradiation in both arsenate-treated and untreated cultures. Prior treatment with arsenate did not affect the lag phase (growth recovery) or the growth time of the wild type strain, regardless whether irradiated or not (S6 Fig). Interestingly, a delay in growth recovery has been reported to contribute to bacterial survival after antibiotic treatment (“tolerance by lag”) [49], which might suggest that an elongated lag phase may also contribute to increased survival after UV irradiation.

In summary, we demonstrated that endogenous prophages contribute to the bactericidal effects of the gyrase inhibitor, ciprofloxacin. The presence of the toxin-encoding gene *tisAB* leads to low ATP levels and a dampened SOS response, resulting in low prophage induction upon *tisAB* expression. Thus, *tisAB* reduces prophage-mediated killing and increases the TisB-associated ciprofloxacin persister fraction. [9]. In the future, it would be interesting to determine whether TisB also affects prophage induction in other species, thereby influencing persister levels.

## Discussion

Bacterial persister cells have been associated with treatment failure and chronic infections, and are considered to constitute a serious health threat [50]. Bacterial persistence increases the likelihood of antibiotic resistance, and a single exposure to DNA damaging agents such as fluoroquinolones has been shown to promote resistance to diverse classes of antibiotics [51,52]. Therefore, it is important to understand how persisters survive treatment with antibacterial agents and how this knowledge can be used to reduce survival and persistence and the risk of the emergence of resistant strains. Different pathways leading to persistence have been described, but usually persister cells are described as metabolically inactive. Consistent with this phenotype, the ATP concentration of bacteria has been reported to be significant for persister cell formation [10,11]. It was proposed that low ATP levels reduce the activity of antibiotic’s targets, thus promoting the formation of persister cells, which survive the treatments due to reduced cellular damage. However, the involvement of ATP in the context of drug-tolerance appears to be more complex because mutation of the ATP synthase can also significantly reduce persistence, despite reduced ATP levels [29,47]. In *Mycobacterium tuberculosis* for example, inhibition of the ATP synthase by bedaquiline interfered with the feedback inhibition of glycolysis, thus increasing both metabolic activity and drug susceptibility [53]. Additionally, the deletion of the *atp* operon in *S*. Typhimurium reduces the ATP concentration but increases NADH formation, which stimulates reactive oxygen species (ROS) production through flavin reductase, ultimately negatively affecting persister cell levels [47].

Similar to the results presented here, other studies have shown a link between prophage induction and regulation of the SOS response. In *E. coli*, *lexA* null mutations are lethal due to uncontrolled inhibition of cell division resulting from derepression of the LexA-repressed *sulA* gene, encoding a cell division inhibitor. In *E. coli*, mutations in *lexA* remain viable in *sulA* mutant backgrounds [54,55]. In contrast, in *S*. Typhimurium LT2, introduction of a *lexA* mutation was not possible in a *sulA* mutant background [56]. However, the authors identified rare survivors which showed the expected constitutive expression of a reporter for the SOS response, indicating loss of LexA. Characterization of these *lexA sulA* isolates revealed loss of one or more of the endogenous prophages harboured by this strain. All isolates showed loss of Fels-2, and some showed additional loss of the Gifsy-1 and Gifsy-2 prophages [56]. These prior observations emphasize the importance of bacteria correctly controlling the SOS response, particularly in the context of our study, where the SOS response is activated by ciprofloxacin. This would also be consistent with the known regulatory systems of many coliphage, which rely on RecA and/or LexA to release prophage repression and activate their lytic life cycles, coupling prophage induction with the host SOS response [57].

It has previously been shown that the levels of DNA damage in persister cells and drug-sensitive bacteria is indistinguishable after fluoroquinolone treatment, and that survival depends on the timing of DNA replication and cell division during the recovery (post-antibiotic) period [32,38]. Delaying the time before growth resumption after fluoroquinolone treatment, *e.g.,* through a period of carbon source starvation prior to transfer to growth-supporting media, resulted in nearly complete survival of treated cultures, and re-growth was dependent upon RecA-dependent recombination repair [38]. An initial strong activation of *tisAB* and hence TisB-mediated decrease in ATP levels as part of the SOS response, would therefore result in growth inhibition and reduce ATP-dependent metabolic activities [23]. In turn, this would provide the necessary time for RecA-dependent repair before resumption of cell division, which would otherwise lead to cell death. Furthermore, binding of RecA, to the DNA is ATP dependent [58,59], suggesting that low ATP levels have an impact on the initiation of the SOS response. Therefore, decreases in ATP levels, as a result of TisB activity, would also prevent a strong induction of the SOS response (Fig 2C), permitting induction of the DNA repair system without activating endogenous prophages. Conversely, an uncontrolled increase in ATP would favor the binding of RecA to damaged DNA, as seen in the *tisAB* mutant as indicated by increased RecA filaments on the DNA (Fig 2A and 2B). Activation of RecA-ATP would thereby result in a strong induction of the SOS response and prophage induction and ultimately lead to prophage-mediated killing and a reduced antibiotic persistence (Fig 4). The *tisAB* mutant is unable to downregulate the SOS response. There may also be a narrow range within which the SOS response must be regulated to avoid inducing prophages, which could lead to bacterial death. Low induction of the SOS response and inefficient repair of damaged DNA could lead to cell death as bacteria attempt to divide. *tisAB* is processed into *tisB* mRNA, and translation can be blocked by the antitoxin IstR-1 through base pairing [60]. Thus, heterogeneous transcription of the antitoxin could also lead to heterogeneous repression of *tisB*, allowing only a small subpopulation to translate *tisB* mRNA into protein. This could explain why only a subpopulation benefits from TisB activity after induction of the SOS response. For example, when istR-1 expression is already low before induction of the SOS response, it could give the bacteria the opportunity to quickly reduce ATP concentration before the DNA repair system is fully activated in conjunction with prophages.

Bacteriophage lysis of the bacterial host at the end of the lytic cycle involves the activity of lysis proteins, including the holins, endolysins and spanins [61–64]. In bacteriophage λ and related lambdoid prophages, such as Gifsy-1 of *S*. Typhimurium, the S holin is a pore-forming protein, allowing the diffusion of the endolysin (R) from the cytosol into the periplasm, where it degrades the cell wall of bacteria. Spanins, such as Rz, are involved in the final stage of lysis, being responsible for the fusion of the inner and outer bacterial membranes, leading to membrane disruption and the release of mature virions [61]. However, excessive expression of lysis genes may trigger premature or incomplete lysis, leading to early cell death and a lower burst size [65]. These genes are activated in Gifsy-1 by an antirepressor (GfoA), which is regulated by RecA and LexA [43]. FACS analysis revealed that the induction of GfoA is much stronger when *tisAB* is deleted (Fig 4D). This is seen as a stronger up regulation of Gifsy-1, which is also consistent with our previous study (see also Fig 4C) [21]. The reduced induction of the antirepressor could be an effect of RecA aggregation in the cytosol, as shown by microscopic imaging (Fig 2), which would prevent RecA from binding to DNA when aggregated. This is further supported by the study of Leinberger *et al*., where overexpression of *tisB* in *E. coli* led to protein aggregation and increased survival after exposure to ciprofloxacin [36]. Furthermore, our microscopic imaging revealed stronger RecA filamentation in the *tisAB* mutant, indicating binding to bacterial DNA [33], which, in turn, depends on ATP availability [59] (Fig 2A and 2B). RecA filamentation could be reduced by lowering the intracellular ATP concentration using arsenate, which was accompanied by an increase in RecA foci formation. Interestingly, despite being upregulated after two hours of ciprofloxacin treatment, ST64B had no significant effect on persister levels [21]. This might be explained by a recent study, which showed that Gifsy-1 inhibits the lytic cycle of ST64B through the activity of a tRNA-cleaving protein encoded by *remN* on Gifsy-1 [22]. This may represent a defense mechanism of Gifsy-1 against other, more rapidly induced prophages, which, unlike Gifsy-1, do not need to express an antirepressor to deactivate the Gifsy-1 repressor.

**Fig 6:**
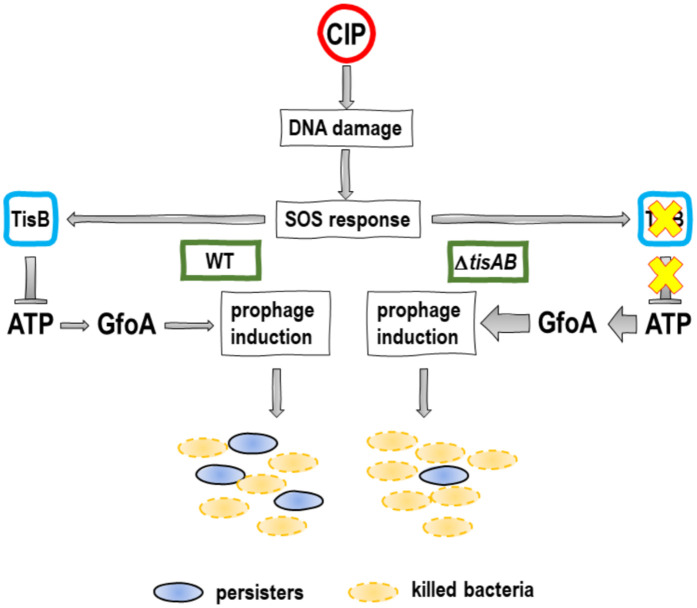
Proposed model of *tisAB*-mediated inhibition of prophages following ciprofloxacin treatment. Treatment with ciprofloxacin (CIP) induces DNA damage, which in turn triggers the bacterial SOS response. In addition to the SOS response, *tisAB* is also activated in the wild type, leading to a reduction in intracellular ATP levels. This ATP reduction results in weaker filamentation of RecA to the DNA, causing a weaker induction of the RecA-dependent antirepressor GfoA and prophages. In contrast, the *tisAB* mutant, which lacks the *tisAB* pathway, is unable to reduce ATP levels, leading to stronger induction of prophages and ultimately reducing the persister fraction.

In addition to ATP depletion, TisB activity was shown to decrease intracellular pH and stimulate the formation of H₂O₂ following treatment with ofloxacin [66]. Both conditions can damage proteins and lead to protein aggregation [67,68]. However, in this study, the crucial factor for increased ciprofloxacin tolerance is the reduced ATP level affecting RecA binding to DNA. Not only was RecA filamentation lower in the wild type compared to the *tisAB* mutant, but it could also be further reduced by the addition of arsenate, indicating ATP-dependent RecA binding to DNA. Furthermore, UV tolerance of the prophage-positive wild type increased when pre-exposed to arsenate, whereas no effect was observed for the prophage-free counterpart. This may suggest an evolutionary development of *tisAB* to protect bacterial cells from prophage-mediated death during DNA damage and the SOS response. It would be interesting to investigate whether other bacterial species with *tisAB* homologues exhibit a similar relationship between *tisAB* activity and prophage induction. This could make *tisAB* also an interesting target for the development of future antibacterial agents to enhance prophage-mediated killing during drug treatment, thereby reducing persister survival and recurring infections. The TA module *hok*/*sok* has been found to reduce the efficiency of T4 plating, decrease plaque size, and lower T4 burst size, presumably by triggering cell death and thus interfering with the dissemination of the lytic bacteriophage T4 [69]. However, bacterial toxins can also be used by prophages to regulate the lysogeny/lytic cycle. In contrast to TisB, which protects the bacterial host from prophage-mediated killing, φ3T_93, a *Bacillus*-infecting prophage, exploits the bacterial TA module MazE/MazF to regulate its propagation [70]. The toxin MazF is activated by YopM, a prophage-encoded protein. This toxin degrades mRNA and therefore reduces prophage-mediated lysis, allowing φ3T_93 to enter into the lysogenic cycle after infection. These examples, whether TisB, Hok, or MazF, highlight complex interactions between prophages and their hosts, involving the activity of bacterial toxins. In this context, it might be understandable why the deletion of RNA-interferase toxins reduced persister cell levels after treatment with fluoroquinolones in accidentally prophage-infected *E. coli* [12–14]. A model of *tisAB*-dependent persister survival after ciprofloxacin treatment is illustrated in Fig 6.

Lytic bacteriophages were used as natural antibacterial agents before the discovery of antibiotics [71]. However, when penicillin and other antibiotics became commercially available, the use of lytic bacteriophages became obsolete. Only recently has the focus shifted back to lytic bacteriophages, considering them as an adjuvant or alternative to antibacterial drugs [72]. This is important because the steadily increasing number of resistant bacteria and the slow development of new antibiotics are forcing us to find new alternatives. A well-studied phenomenon is the phage-antibiotic synergy effect (PAS), where the combination of antibiotics with lytic bacteriophages can improve the efficiency of antibiotics [73,74]. However, one drawback of PAS is that only strain-specific lytic bacteriophages can be used to enhance antibiotic killing, and these must be isolated and purified before treatment. A more efficient alternative to classical PAS is temperate PAS (tPAS) [75]. In this case, prophages already integrated into the bacterial chromosomes are exploited to increase drug-mediated killing. Interestingly, tPAS represents a novel area of research with substantial scope for further investigation. RecA appears to be essential for prophage-mediated killing in tPAS [75]. Therefore, it would be interesting to study the effect of *tisAB* on tPAS to determine whether *tisAB* could further enhance the effect of tPAS by increasing the induction of the SOS response and prophages, reducing persister cell formation and preventing the evolution of resistance through tolerance/persistence. This approach could be particularly interesting considering that top-priority pathogens, including *Acinetobacter*, *E. coli*, *Shigella*, and *Pseudomonas*—defined by the World Health Organization (WHO)—are developing resistance to antibiotics at an alarming rate [76]. However, it is also known that these pathogens usually harbour a number of prophages, which could be exploited and activated to kill the bacteria [76,77]. This may also shed light on the possibility that TA modules, such as TisB/IstR-1, have evolved to protect bacteria against a strong induction of prophages and subsequent cell death.

In summary, our study sheds new light on the interplay between ATP and persistence mechanisms related to DNA damage, prophage induction, and the role of TisB. Through its interference with ATP production, TisB limits prophage induction and subsequent bacterial killing and thereby increases antibiotic persistence to DNA-damaging agents such as fluoroquinolones.

## Materials and methods

### Media and antibiotics

All experiments were conducted in Lennox Broth (LB) purchased from Carl Roth. Ciprofloxacin (Sigma-Aldrich) used for the persister assays were applied at four-fold the MIC (1 µg/ml, see below).

### Bacterial strains

A summary of all used strains can be found in Table A in S1 Text. The wild type background used in this study is *Salmonella enterica* serovar Typhimurium strain ATCC 14028s [29].

The strains harbouring deletions of the Gifsy-1 (ΔGifsy-2::*kan*), Gifsy-2 (ΔGifsy-2::*cat*), and ST64B (ΔST64B::*kan*) prophage have previously been described [71]. Deletion of Gifsy-3 was performed using the λ Red mutagenesis/gene replacement method as previously described [78]. The kanamycin resistance gene was amplified from pKD4, using the primers indicated in Table B in S1 Text. PCR products were introduced by electroporation into electrocompetent bacteria harbouring plasmid pSIM6, and which had been heat-induced to express λ Red recombinase prior to preparation of electrocompetent cells [78]. After electroporation, the bacteria were plated onto LB agar plates containing kanamycin for selection of transformants. The successful deletion of Gifsy-3 was verified by PCR using the primer pairs pp599-ppR25 and pp587-pp549. PCR-positive, putative mutant strains were sequenced to further confirm deletion of Gifsy-3. The strain harbouring the deletion in Gifsy-3 (MA14253), was used as a donor for P22 transductions into other strains with selection for kanamycin resistance using standard protocols. Construction of additional mutants were as described above using plasmid pKD4 as template for the amplification of the kanamycin resistance cassette [78].

To construct the prophage-free variant of our wild type strain (8640), we prepared P22 lysates from donor strains containing a kanamycin resistance cassette in place of the corresponding prophage. The deletion of the respective prophage was then sequentially introduced into our wild type 8640 using the P22 lysates with selection for kanamycin resistance. To avoid carry over of P22 infected transductants, strains were first purified by screening on Green plates [79], and successful deletion/gene replacements were verified via PCR. The strains were subsequently transformed with plasmid pCP20, encoding for a FLP recombinase, to remove the kanamycin resistance cassette [78,80]. After confirmation of the successful elimination of the antibiotic resistance, the strains were incubated at 37°C for pCP20 removal. Loss of pCP20 was verified by screening the strains for lack of growth on ampicillin and chloramphenicol plates at 28°C.

To construct the recA-mScarlet strains, mScarlet-I from the plasmid pMRE135 [81] was amplified using the primers LinkScarlet and mScarlet-HindIII-Rev to introduce the restriction sites for EcoRI and HindIII as well as the linker sequence (gcgagcatgaccggcggccagcagatgggccgcattcgcattcat) at the N-terminus of mScarlet-I. Subsequently, the fragment was cloned into p2795 [82]. Successful ligation was confirmed by PCR and sequencing. The resulting plasmid pSeb5 was used as a template to replace the stop codon TAA of *recA* with the Linker-mScarlet-I construct to obtain a recA-mScarlet-I fusion. The further approach was as described above. To replace *gfoA* with mCherry, pSeb1 was generated as described for pSeb5. mCherry was amplified from pFCcGi [83], cloned into p2795 [82], and used as a template.

### Persister assay/ UV killing assay

6 ml LB were inoculated with one colony coming from a LB plate, which was streaked out the day before and incubated at 37°C. The culture was further incubated at 37°C while shaking at 200 rpm. After the bacteria reached the appropriate optical density (OD_600_ = 0.5; OD_600_ = 1.0; OD_600_ = 2.0; over-night), 5 x 10^6^/ml were transferred into 6 ml fresh LB. Subsequently, an aliquot was removed to determine the CFU before addition of 1 µg/ml ciprofloxacin, representing four-fold the MIC concentration (4X MIC), previously determined at 0.0625 µg/ml. Note that the MIC assays are performed in 1:1 dilution steps, hence 4X MIC corresponds to the ciprofloxacin concentration three dilution steps prior to the MIC (1 µg/ml), rather than a four-fold concentration of the MIC (0.25 µg/ml). At the indicated time points, samples were removed, washed and resuspended in 1X PBS before plating to LB agar plates. After incubation at 37°C, colonies were counted to determine the survival.

For the UV killing assays, the bacteria were incubated to OD_600_ = 0.5 and diluted to 5 x 10^6^/ml before adding the bacterial suspension into a petri dish for UV irradiation using a Gel Doc XR + Gel Documentation System (BioRad). The petri dish was placed in the chamber of the transilluminator and irradiated with an array of six eight-watt UV lamps. To damage the DNA, the bacteria were exposed to UV-B radiation at a wavelength of 280 nm to 300 nm, coming directly from below where the petri dishes were placed. The bacteria were exposed to energy, which is the total energy divided by the surface area exposed to UV, and this is indicated in the respective figures as mJ/cm^2^. After irradiation, the bacteria were plated onto LB plates and incubated overnight at 37°C for colony counting the next day. To reduce the intracellular ATP concentration, bacteria were pre-incubated with 0.5 mM sodium arsenate for 20 min at 37°C with shaking prior to the UV killing assays.

### Anti-repressor induction

The strains SB287 and SB288 were incubated under the same conditions as for the persister assays and then treated with ciprofloxacin. At the indicated time points, bacteria were washed, resuspended in 1 × PBS, and analyzed via FACS for mCherry expression using a CytoFLEX (Beckman Coulter). For the recovery phase, bacteria were incubated in LB at 37 °C. At least 500,000 events were recorded. Strains SB22 and SB536 were treated identically to assess GFP fluorescence.

### Confocal microscopy and image analysis

The strains SB448 and SB451 carrying the recA-mScarlet-I construct were incubated as for the persister assay to mid-log. phase, either in LB only or with 20 min pre-incubation with 0.5 mM sodium arsenate. Subsequently, 1 µl was transferred onto LB soft agarose pads (1.5% low melting agarose), which were held in place by silicone isolators A/A purchased from Electron Microscopy Sciences (size 13 mm dia x 0.1 mm depth). The agarose pads were covered using covers glasses from Zeiss (18 mm x 18 mm; 0.170 + /- 0.005 mm). Afterwards, the microscopic slides were incubated for two hours at 37°C and then prepared for imaging. Confocal microscopy was performed at the Service Unit Microscopy of the Veterinary Centre for Resistance Research (TZR) with an inverted Leica Stellaris 8 FALCON confocal microscope, equipped with a 405 nm laser, a white light laser (WLL, 440 – 790 nm), an acousto-optical beam splitter and Power HyD detectors. The microscope was operated by LAS X version 4.5.0.25531. Images were acquired with a HC PL APO 63x/1.40 oil immersion objective. To generate large tile scans for automated image analysis, excitation of mScarlet-I was performed at 570 nm using the WLL set to 0.15% intensity, applying a unidirectional resonant scanning mode with 8 line accumulations at 8000 Hz scan speed. Fluorescence emission was collected between 575 – 650 nm using a HyD S detector in intensity mode (gain 2.5) and the pinhole set to one airy unit. A brightfield image was acquired simultaneously using a Trans-PMT detector. A zoom of 2 was applied resulting in a pixel size of 82 nm (@ 1120 x 1120 pixel) and images consisting of 18 optical sections with a z-step size of 333 nm were acquired as 3 × 3 tile scans. For high resolution images shown in Fig 2, mScarlet-I was excited as above but with 0.20% intensity applying the unidirectional standard scanning mode with 3 line averages at 600 Hz scan speed. Fluorescence emission was also collected between 575 – 650 nm using a HyD S detector in intensity mode (gain 10.1) and the pinhole set to one airy unit. A brightfield image was acquired simultaneously using a Trans-PMT detector. A zoom of 2 was applied resulting in a pixel size of 62 nm (@ 1496 x 1496 pixel) and images consisting of 18 optical sections with a z-step size of 333 nm were acquired. Post‐processing of the mScarlet channel was performed using LAS X LIGHTNING with ‘Mounting Medium’ set to Water (RI = 1.33) and all other settings left as default and subsequently stitched together in LAS X. Finally, Fiji/ImageJ [74] was used to merge brightfield images with images from the mScarlet-I channel and to linearly adjust brightness and contrast. To quantify the number of bacteria with RecA foci and filaments, projections of images stacks were generated (maximum intensity projections of the RecA channel and extend depth of field projections comprising approx. 5 in-focus sections of the transmission channel) and cropped to 3000 x 3000 pixel using Fiji/ImageJ. Next, bacteria were segmented based on the projected transmission channel using Omnipose (version 1.0.7.dev20) with the provided pretrained ‘bact_phase_omni’ model [84] after downscaling the images to 1000 x 1000 pixel to match the size of the bacteria to the training data of the pretrained model. Resulting mask images were rescaled to 3000 x 3000 pixel and together with projections of the RecA channel loaded into CellProfiler (version 4.2.6) for further analysis. The used analysis pipeline is schematically depicted in S6 Fig. In brief, after rescaling intensities, smoothing and subsequent enhancement of the structures, RecA positive objects were identified. Next, after removing bacteria touching image borders, remaining bacterial masks were separated into RecA-positive and RecA-negative bacteria. The size and shape of the all RecA objects was measured and these objects were separated based on eccentricity (< 0.9 = foci, ≥ 0.9 = filaments). RecA-positive bacteria were related with the filaments and assigned to the class `bacteria with RecA filaments’ if they contained at least 1 filament object. All remaining RecA-positive bacteria were assigned to the class `bacteria with RecA foci'. The number of bacteria in each class was exported for final data analysis. For quality control purposes outlines of the bacteria in the different classes were overlayed on the RecA images and saved. Samples were randomized and blinded during image acquisition and analysis.

### ATP concentration

The ATP levels in *S.* Typhimurium at different growth phases or after incubation with sodium arsenate were determined using the BacTiter-Glo Microbial Cell Viability Assay Kit (Promega) and performed according to the manufacturer’s protocol. The wild type strain was incubated in LB at 37°C with aeration to an OD_600_ = 0.5, diluted to 10^7^/ml, and further incubated for 20 min with the indicated arsenate concentrations. Subsequently, the ATP concentrations were determined in a 96-well plate using a BioTek Synergy HTX plate reader, and the luminescence was normalized to the CFU. For comparison, an untreated culture was used as control. The same procedure was performed to measure ATP at different growth phases (OD_600_ = 0.5; OD_600_ = 1.0; OD_600_ = 2.0 and overnight).

### Membrane potential

The *Bac*Light Bacterial Membrane Potential Kit from ThermoFisher was used to determine the membrane potential of the wild type and Δ*atp* operon deletion mutant with modifications of the manufacturer’s protocol to improve permeabilization of the membrane [85]. The wild type and Δ*atp* operon mutant were grown to the indicated optical densities, 1 ml were removed and centrifuged for 5 min with 12000 x g. Subsequently, the bacteria were resuspended in the same volume of membrane potential buffer (10 mM Tris-HCl; 1 mM EDTA; 10 mM glucose) and diluted to 10^6^/ml. 200 µl of the dilution was then transferred into a well of a 96 well plate and 30 µM DiOC_2_ was added to all samples. Additionally, a control with 10 µM carbonyl cyanide m-chlorophenyl hydrazone (CCCP) to decrease the membrane potential was prepared in the same manner. The membrane potential was determined by cytometric flow analyses with excitation at 482 nm and emission at 497 nm and 610 nm using the CytoFLEX flow cytometer (Beckmann/Coulter). The same protocol was used to determine the membrane potential at different growth phases.

### Real-Time PCR

Real-Time PCR was used to quantify the induction of prophages, *tisB* and *sulA*. After two hours of treatment with 1 µg/ml ciprofloxacin at 37°C with aeration, total RNA was extracted using the RNeasy Mini Kit (Qiagen) according to the manufacturer’s protocol. In addition, the bacteria were washed with 1x PBS and resuspended in drug-free LB before RNA extraction (recovery phase). After extraction, the RNA was transcribed into cDNA using 2 µl of 50 µM random hexamer primers, 2 µl of 10 mM dNTPs (Invitrogen), 1 µl of 200 U/µl reverse Transcriptase (Thermo Scientific), and 0.5 µl of 40 U/µl RiboLock RNase Inhibitor (Thermo Scientific). 5 ng of the resulting cDNA preparations and SYBR Green Master Mix (Thermo Fisher Scientific) were added into a 96-well plate. The primers used for real-time PCR are provided in Table C in S1 Text. The signal was normalized to the housekeeping gene *trpA* [21,86]. The genes used to monitor prophage induction are as follows: STM2605 (a head-tail preconnector protein), STM1048 (a prophage-encoded host specificity protein, homologous to the phage lambda protein J and a component of the tail tip complex), SspH1E3 (a ubiquitin-protein ligase effector protein specifically encoded by the Gifsy-3 prophage [19]), and sb41 (a gene specific to ST64B with unknown function, which is activated upon induction of the ST64B prophage [42]). All of these genes are encoded within their respective prophages.

To determine the lysis efficiency of 11326/SB149 and SB522/SB523, we treated the bacteria as described above. To obtain the DNA, the bacteria were harvested at the indicated time points and washed three times with 1 × PBS to remove prophages from the supernatant. Subsequently, the pellet was resuspended in water and heated for 5 minutes at 99 °C. The lysates were diluted 1:10 and used for qPCR.

### Plaque Assay

The bacterial strains MS1487 and SB521 were incubated under conditions identical to those used in the persister assays and subsequently treated with 1 µg/ml ciprofloxacin. At the indicated time points, 1 ml of culture was removed, centrifuged, and the supernatant transferred to a new Eppendorf tube. Chloroform (50 µl) was added, the mixture was vortexed, and 2 µl of the resulting solution were spotted onto soft agar (0.5%) overlaid on solid LB agar plates. As a prophage-free strain, we used MS480, kindly provided by Sargen and Helaine [22].

### Scan-Lag

The Scan-Lag system was used [87] to measure the growth resumption of logarithmic growing bacteria before and after UV exposure either with or without 0.5 mM sodium arsenate. *S.* Typhimurium was prepared as for the UV killing curves after growth in LB at 37°C with aeration, and subsequently irradiated with UV (5 mJ/cm^2^) and plated onto LB agar plates. The plates were incubated overnight on an array of EPSON V370 flatbed scanners and colony appearance was monitored by taking pictures in 15-minute intervals. Image analysis was performed as described in [87], using software written in MATLAB R2014a.

## Supporting information

S1 Fig Survival upon drug treatment correlates with the ATP level and membrane potential. A) *S*. Typhmurium (ATCC 14028) was incubated to the indicated cell density before treatment with four-fold the MIC of ciprofloxacin (1 µg/ml). B) In parallel, the relative wild type ATP level was determined, in which the luminescence signal correlates with the ATP level. The generated luminescence signal was normalized on the number of bacteria. C) Determination of the membrane potential at different optical densities using DiOC2. To artificially reduce the membrane potential, the bacteria were pre-treated with 15 µM CCCP. At least three independent experiments were performed for each assay.
(TIF)

S2 Fig Persister assays with Δ*tisB* and the complemented *tisAB* mutant. A) The bacteria (wild type = 8640, Δ*tisAB *= 10752, Δ*tisB* = SB493) were incubated to mid-log phase and treated with 1 µg/ml ciprofloxacin. B) Treatment as in A), but with the chromosomally complemented *tisAB* strain (8640 tisAB+ = SB494, Δ*tisAB tisAB*+ = SB499). At least three independent experiments were performed for each assay.
(TIF)

S3 Fig ATP determination of Δ*tisB*, the complemented *tisAB* strain, and the prophage-free variants, as well as the induction of *tisB.* A) The bacteria (cntrl = SB494, Δ*tisAB tisAB*+ = SB499, Δ*tisB* = SB493) were incubated to mid-log phase and treated with 1 µg/ml ciprofloxacin, indicated in the figure as + CIP. B) Treatment as in A), but with the prophage-free strains. The results were normalized to the wild type (in A) to SB494 or in B) to 11126). C) Transcriptional upregulation of *tisB* following ciprofloxacin treatment in the wild type (8640) and the respective prophage-free variant (11126). Data are presented as means ± standard deviation from at least three independent experiments. Significance was calculated with an unpaired Student’s t-test.
(TIF)

S4 Fig Persister assays and membrane potential determination. A) Persister assays of the *S*. Typhimurium wild type (8640) either with or without resident prophages (11126). Both strains were incubated to the stationary phase (overnight) and subsequently exposed to four-fold the MIC of ciprofloxacin. B) Determination of the membrane potential of the wild type (grey bars) and the *atp* operon mutant (9200, red bars) during exponential growth. The percentage of fluorescence positive bacteria is illustrated as relative fluorescence unit (RFU). CCCP was used to artificially reduce the membrane potential as a control. Significant differences were calculated using the unpaired student`s t-test (two tailed). Three independent experiments were conducted.
(TIF)

S5 Fig GFP translation during and after ciprofloxacin treatment. Bacterial strains SB22 (wild type *rpsM*::*gfp*) and SB536 (Δ*tisAB rpsM*::*gfp*) were treated with 1 µg/ml ciprofloxacin and harvested at the indicated time points for FACS analysis to measure GFP fluorescence. For the recovery phase, the bacteria were incubated in LB medium at 37°C. A total of 500,000 events were recorded per sample. Three independent experiments were conducted.
(TIF)

S6 Fig ScanLag of *S*. Typhimurium before and after exposure to UV The wild type was incubated to the mid. log. phase and subsequently exposed to UV. To determine the lag phase of the bacteria, the bacterial survivors were plated on LB plates and the growth was monitored using flatbed scanners. Afterwards, the average lag phase (colony appearance time) and the growth rate (growth time) were calculated. Where indicated, bacteria were pre-treated with 0.5 mM arsenate. Significant differences were calculated using the unpaired student`s t-test (two tailed). At least three independent experiments were performed.
(TIF)

S7 Fig CellProfiler analysis scheme. (TIF)

S1 Text Strains, Primers, Plasmids & Suppl. Fig. Legends. Table B: Plasmids used in this study. Table C: Primer used in this study (mutagenesis, cloning and real-time PCR).
(DOCX)

S1 Data Raw data of Figs 1 to 5. (XLSX)

S2 Data Raw data of S1 to S6 Figs. (XLSX)
