# Supplementary figures and images for: TisB enables antibiotic tolerance in Salmonella by preventing prophage induction through ATP depletion
Source: PLoS Pathog. 2025 Sep 22;21(9):e1013498. doi: 10.1371/journal.ppat.1013498 (PMC12626290; doi:10.1371/journal.ppat.1013498)

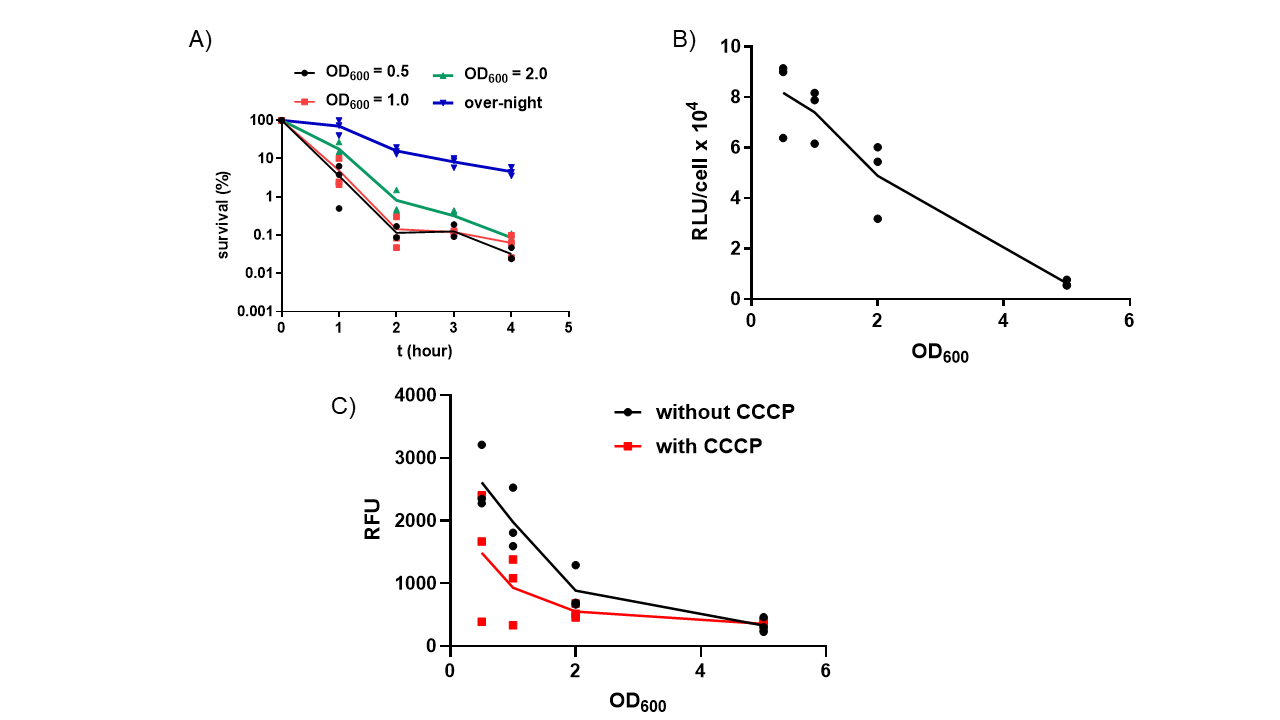

Supplement: S1 Fig — A) S. Typhmurium (ATCC 14028) was incubated to the indicated cell density before treatment with four-fold the MIC of ciprofloxacin (1 µg/ml). B) In parallel, the relative wild type ATP level was determined, in which the luminescence signal correlates with the ATP level. The generated luminescence signal was normalized on the number of bacteria. C) Determination of the membrane potential at different optical densities using DiOC2. To artificially reduce the membrane potential, the bacteria were pre-treated with 15 µM CCCP. At least three independent experiments were performed for each assay. (TIF) [file ppat.1013498.s001.tif]

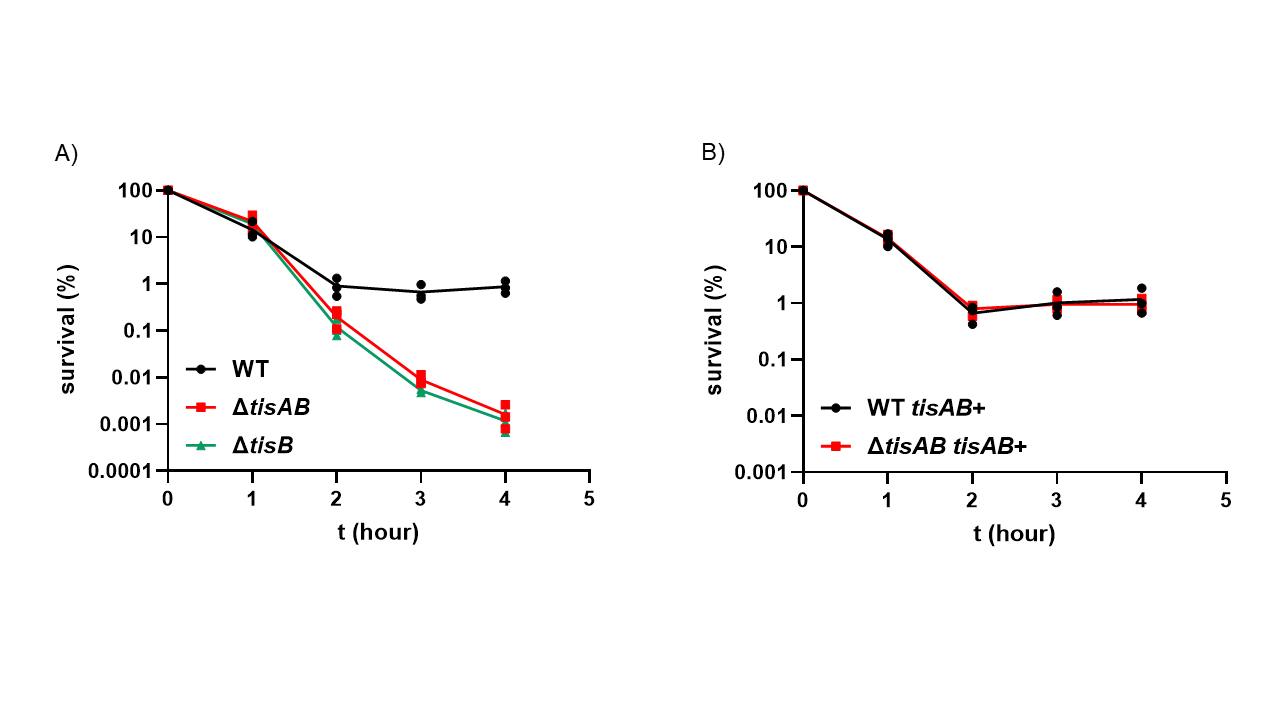

Supplement: S2 Fig — A) The bacteria (wild type = 8640, ΔtisAB = 10752, ΔtisB = SB493) were incubated to mid-log phase and treated with 1 µg/ml ciprofloxacin. B) Treatment as in A), but with the chromosomally complemented tisAB strain (8640 tisAB+ = SB494, ΔtisAB tisAB+ = SB499). At least three independent experiments were performed for each assay. (TIF) [file ppat.1013498.s002.tif]

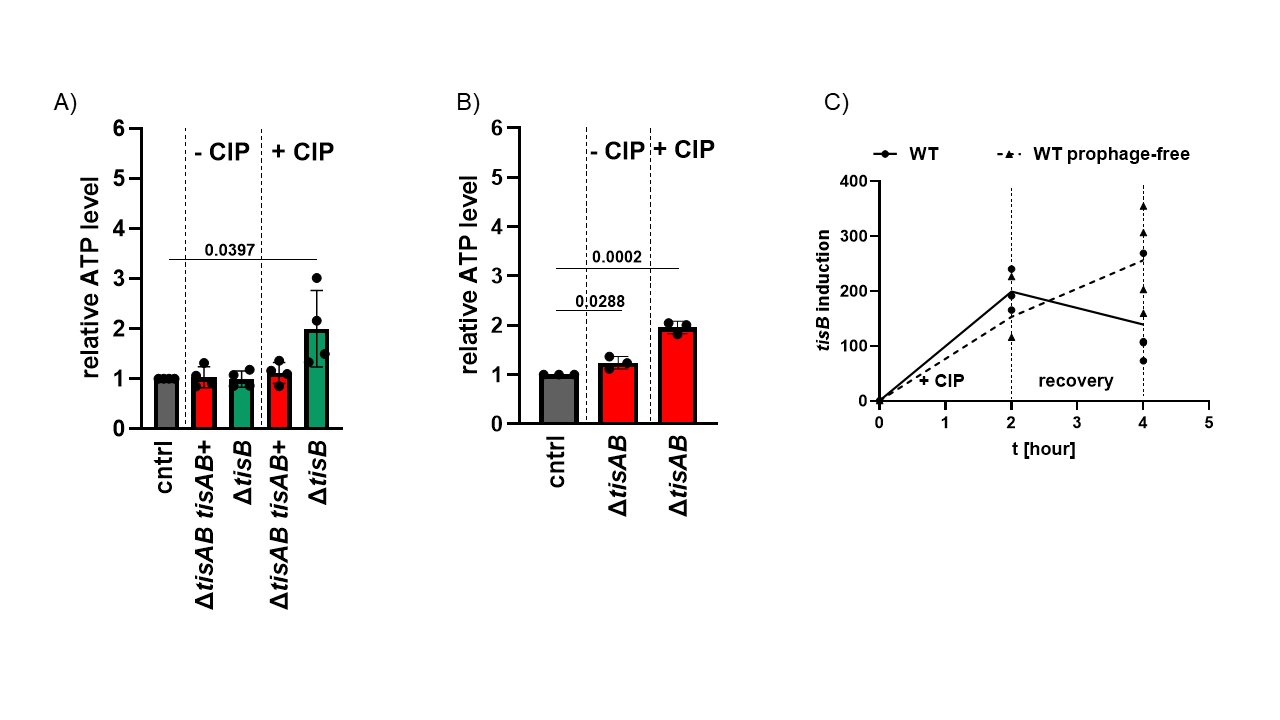

Supplement: S3 Fig — A) The bacteria (cntrl = SB494, ΔtisAB tisAB+ = SB499, ΔtisB = SB493) were incubated to mid-log phase and treated with 1 µg/ml ciprofloxacin, indicated in the figure as + CIP. B) Treatment as in A), but with the prophage-free strains. The results were normalized to the wild type (in A) to SB494 or in B) to 11126). C) Transcriptional upregulation of tisB following ciprofloxacin treatment in the wild type (8640) and the respective prophage-free variant (11126). Data are presented as means ± standard deviation from at least three independent experiments. Significance was calculated with an unpaired Student’s t-test. (TIF) [file ppat.1013498.s003.tif]

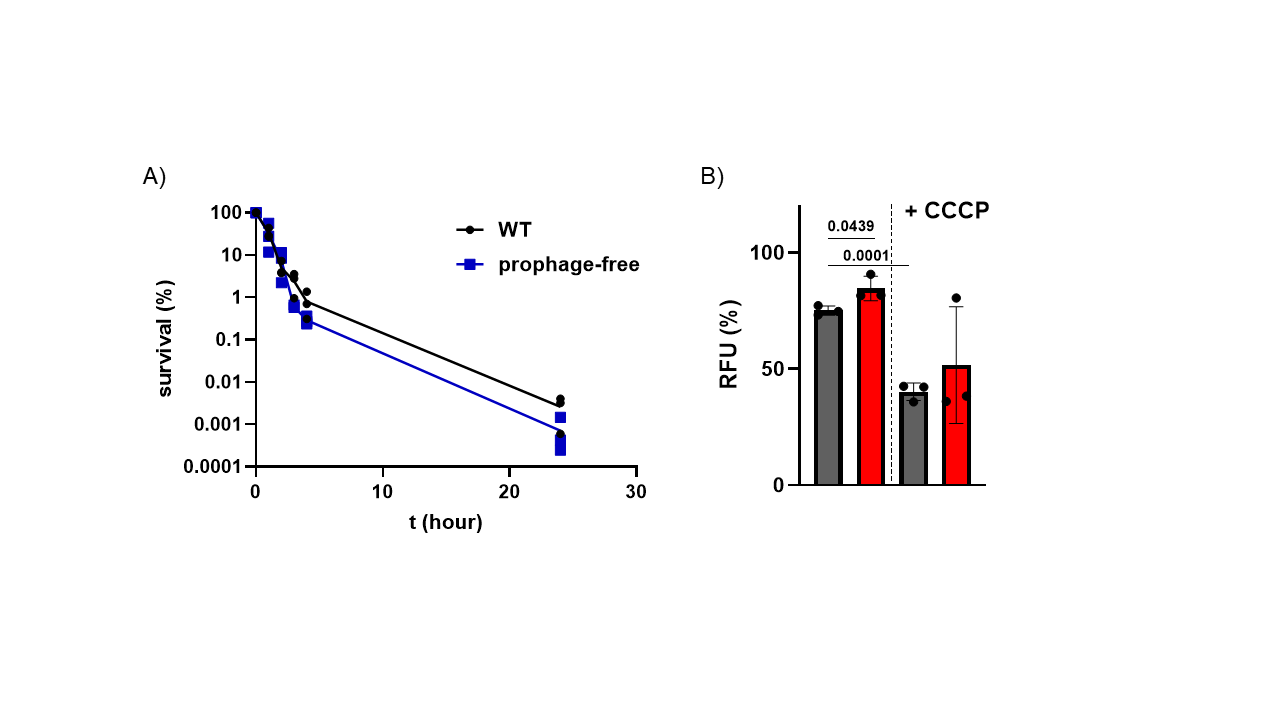

Supplement: S4 Fig — A) Persister assays of the S. Typhimurium wild type (8640) either with or without resident prophages (11126). Both strains were incubated to the stationary phase (overnight) and subsequently exposed to four-fold the MIC of ciprofloxacin. B) Determination of the membrane potential of the wild type (grey bars) and the atp operon mutant (9200, red bars) during exponential growth. The percentage of fluorescence positive bacteria is illustrated as relative fluorescence unit (RFU). CCCP was used to artificially reduce the membrane potential as a control. Significant differences were calculated using the unpaired student`s t-test (two tailed). Three independent experiments were conducted. (TIF) [file ppat.1013498.s004.tif]

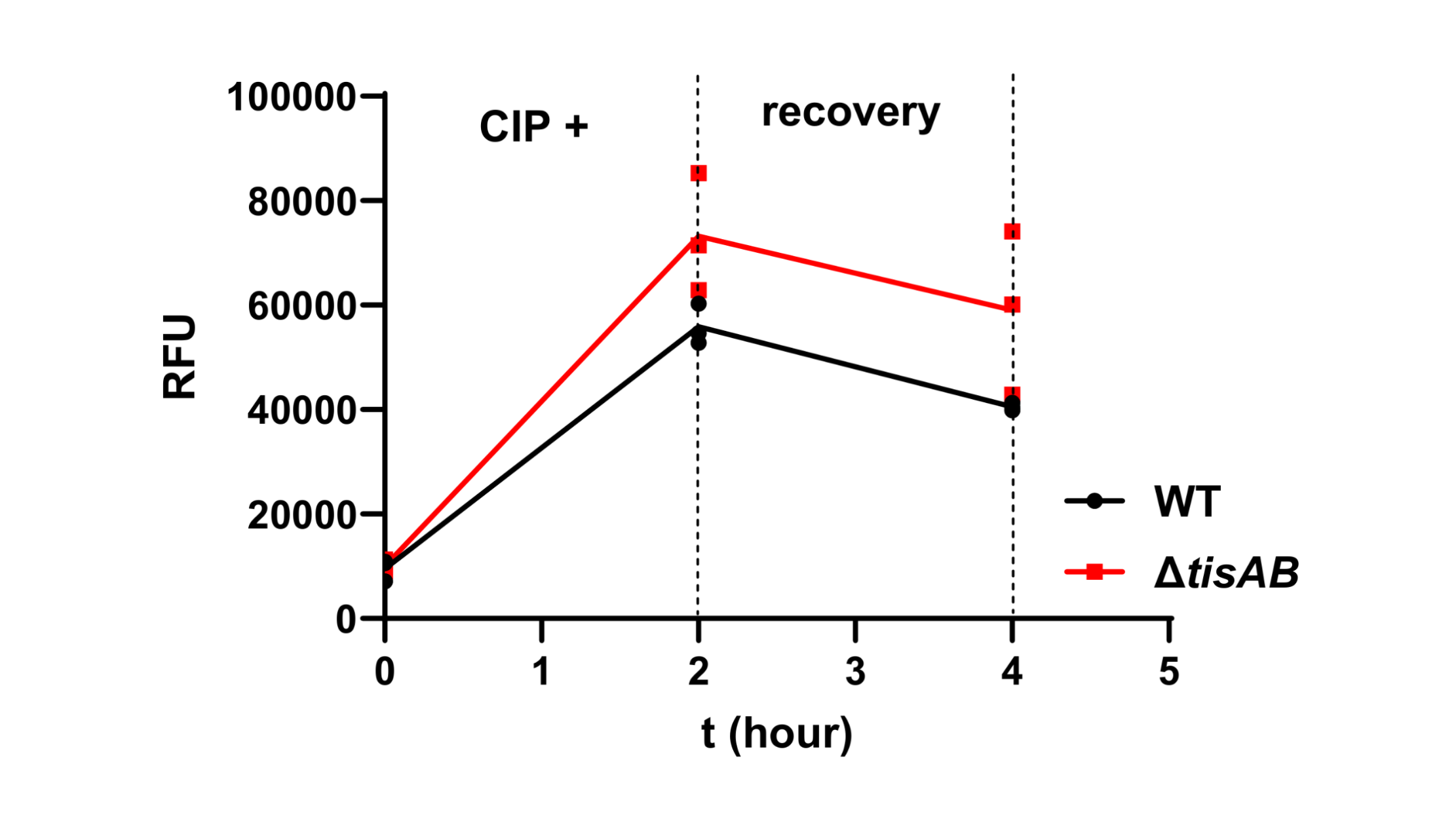

Supplement: S5 Fig — Bacterial strains SB22 (wild type rpsM::gfp) and SB536 (ΔtisAB rpsM::gfp) were treated with 1 µg/ml ciprofloxacin and harvested at the indicated time points for FACS analysis to measure GFP fluorescence. For the recovery phase, the bacteria were incubated in LB medium at 37°C. A total of 500,000 events were recorded per sample. Three independent experiments were conducted. (TIF) [file ppat.1013498.s005.tif]

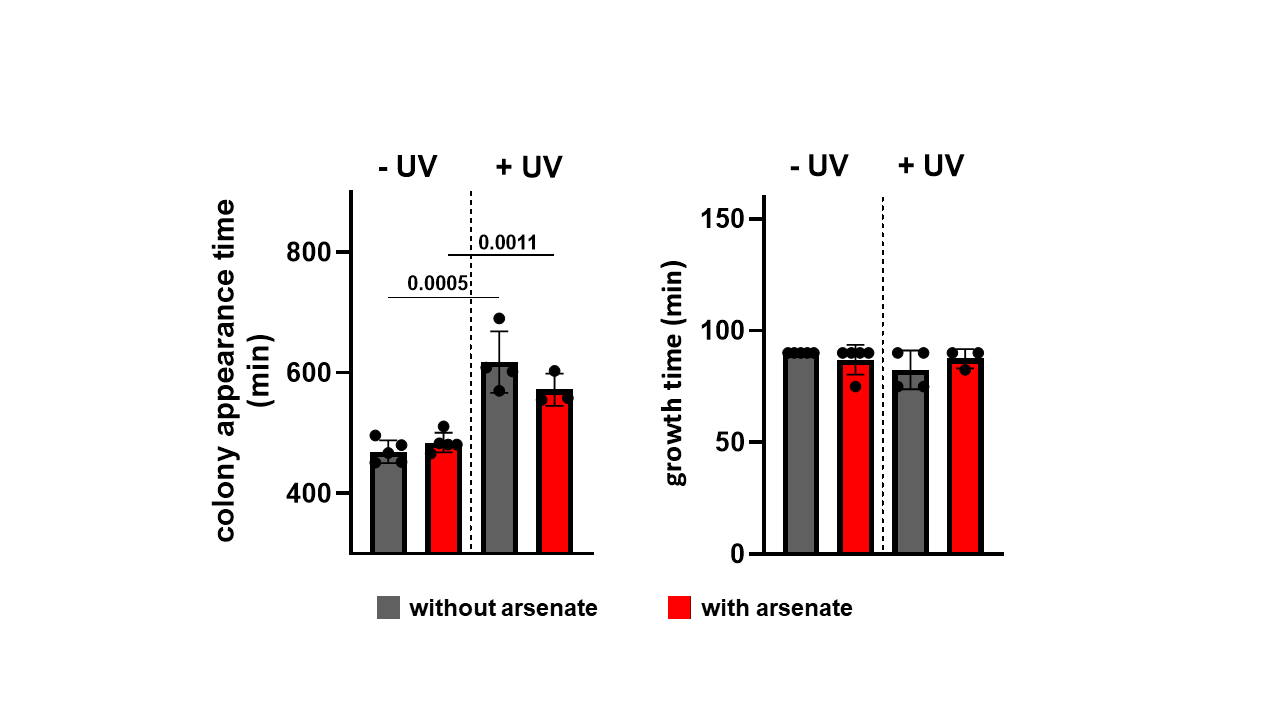

Supplement: S6 Fig — The wild type was incubated to the mid. log. phase and subsequently exposed to UV. To determine the lag phase of the bacteria, the bacterial survivors were plated on LB plates and the growth was monitored using flatbed scanners. Afterwards, the average lag phase (colony appearance time) and the growth rate (growth time) were calculated. Where indicated, bacteria were pre-treated with 0.5 mM arsenate. Significant differences were calculated using the unpaired student`s t-test (two tailed). At least three independent experiments were performed. (TIF) [file ppat.1013498.s006.tif]

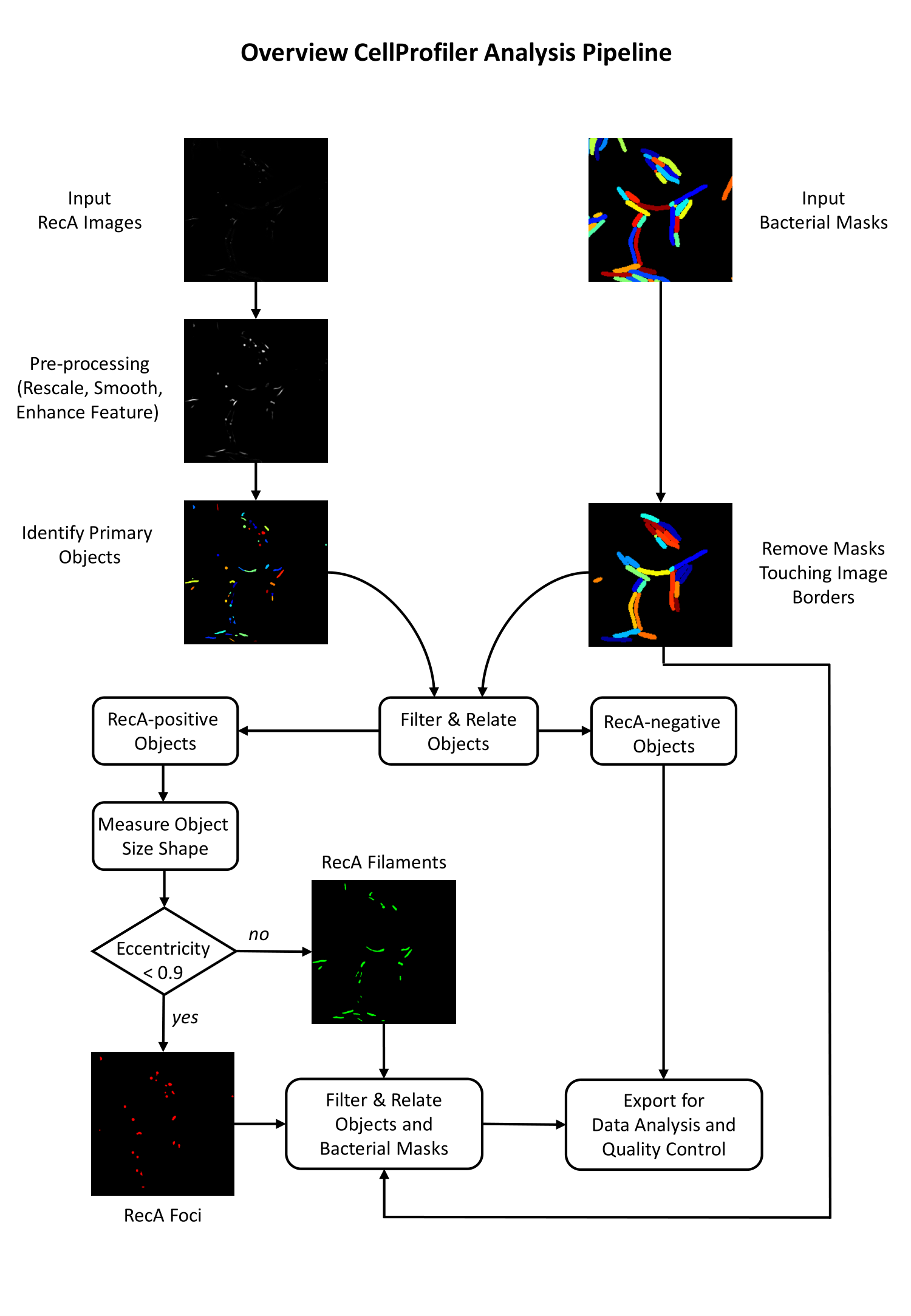

Supplement: S7 Fig — (TIF) [file ppat.1013498.s007.tif]
