# Supplementary material for: TisB enables antibiotic tolerance in Salmonella by preventing prophage induction through ATP depletion
Source: PLoS Pathog. 2025 Sep 22;21(9):e1013498. doi: 10.1371/journal.ppat.1013498 (PMC12626290; doi:10.1371/journal.ppat.1013498)
Supplement: S1 Text — Table B: Plasmids used in this study. Table C: Primer used in this study (mutagenesis, cloning and real-time PCR). (DOCX) [file ppat.1013498.s008.docx]

**Supplementary data**

| **Table A**: Strains used in this study | | |
| --- | --- | --- |
| Strain | Relevant Features* | Reference/Source |
| JS215 | *S*. Typhimurium ATCC 14028 ΔGifsy-1::*kan* | [1] |
| JS223 | *S*. Typhimurium ATCC 14028 ΔGifsy-2::*cat* | [2] |
| MA14253 | *S*. Typhimurium ATCC 14028 ΔGifsy-3::*cat* | This study |
| MA7549 | *S*. Typhimurium ATCC 14028 Fels-1[-] Fels-2[-] Gifsy-1[-] Gifsy-2[-] Gifsy-3[-] ΔST64B::kan | [3] |
| 8640 | *S.* Typhimurium ATCC 14028 wildtype, virulent NalR | [4] |
| 9200 | *S.* Typhimurium ATCC 14028 wildtype, virulent Nal^R^ Δ*atp IBEFHAGDC* | [4] |
| 10752 | *S.* Typhimurium ATCC 14028 wildtype, virulent Nal^R^ Δ*tisAB* | This study |
| 11126 | *S.* Typhimurium ATCC 14028 wildtype, virulent NalR ΔGifsy-1 ΔGifsy-2 ΔGifsy-3 ΔST64B | [5] |
| 11326 | *S.* Typhimurium ATCC 14028 wildtype, virulent NalR ΔGifsy-2 ΔGifsy-3 ΔST64B | This study |
| 11588 | *S.* Typhimurium ATCC 14028 wildtype, virulent Nal^R^ ΔGifsy-1 ΔGifsy-2 ΔGifsy-3 ΔST64B Δ*tisAB* | This study |
| SB22 | *S.* Typhimurium ATCC 14028 wildtype, virulent Nal^R^ rpsM::gfp CAM^R^ | [6] |
| SB536 | *S.* Typhimurium ATCC 14028 wildtype, virulent Nal^R^ Δ*tisAB* rpsM::gfp CAM^R^ | This study |
| SB149 | *S.* Typhimurium ATCC 14028 wildtype, virulent NalR Δ*SRRz* ΔGifsy-2 ΔGifsy-3 ΔST64B | This study |
| SB287 | *S.* Typhimurium ATCC 14028 wildtype, virulent NalR *gfoA*-*dinI*::*mCherry* ΔGifsy--2 ΔGifsy-3 ΔST64B | This study |
| SB288 | *S.* Typhimurium ATCC 14028 wildtype, virulent NalR *gfoA*-*dinI*::*mCherry* ΔGifsy--2 ΔGifsy-3 ΔST64B Δ*tisAB* | This study |
| SB448 | *S.* Typhimurium ATCC 14028 wildtype, virulent NalR, *recA*-mScarlet-I | This study |
| SB451 | *S.* Typhimurium ATCC 14028 wildtype, virulent NalR Δ*tisAB, recA*-mScarlet-I | This study |
| SB493 | *S.* Typhimurium ATCC 14028 wildtype, virulent Nal^R^ Δ*tisB::kan* | This study |
| SB494 | *S.* Typhimurium ATCC 14028 wildtype, virulent Nal^R^ Kan^R^ | This study |
| SB499 | *S.* Typhimurium ATCC 14028 wildtype, virulent Nal^R^ Δ*tisAB tisAB*+ (chromosomally complemented) | This study |
| MS1487 | *S.* Typhimurium ATCC 14028 wildtype, virulent ΔGifsy-2 ΔGifsy-3 ΔST64B | [7] |
| SB521 | *S.* Typhimurium ATCC 14028 wildtype, virulent Δ*tisAB* ΔGifsy-2 ΔGifsy-3 ΔST64B | This study |
| SB522 | *S.* Typhimurium ATCC 14028 wildtype, virulent NalR Δ*tisAB* ΔGifsy-2 ΔGifsy-3 ΔST64B | This study |
| SB523 | *S.* Typhimurium ATCC 14028 wildtype, virulent NalR Δ*tisAB* Δ*SRRz* ΔGifsy-2 ΔGifsy-3 ΔST64B | This study |
| * Abbreviations: Nal, nalidixic acid; *kan*, kanamycin resistance; *cat*, chloramphenicol resistance | | |

**Table B**: Plasmids used in this study

| Plasmids | genotype | Resistance | source |
| --- | --- | --- | --- |
| pKD4 | FRT-kan-FRT | Amp^R^ , Kan^R^ | [8] |
| pSIM6 | pSC101 *repA^ts^* , λ Red recombinase | Amp^R^ | [9] |
| pSeb1 | mCherry + FRT-*aph*-FRT |  | This study |
| pSeb5 | Linker-mScarlet-I + FRT-*aph*-FRT |  | This study |

* Abbreviations: Amp, ampicillin resistance; Kan, kanamycin resistance

| **Table C**: Primer used in this study (mutagenesis, cloning and real-time PCR) | | |
| --- | --- | --- |
| Primer | Sequence | Target |
| TISBH1P1 | GGTACGTCGGCTGGCAGTGCTCCTTAACCACAGGAGACGCGTATGTGTAGGCTGGAGCTGCTTCGA | *tisB* |
| TISBH2P2 | GTTGCCCGCTCCCCTTCGGTGCGGCTTGAATCTGAATTACTTAAGCATATGAATATCCTCCTTAG | *tisB* |
| TISBF | ACAGCTGGCAACAGGCAGCG | *tisB* |
| TISBR | CGAAAGACCAGCGGATAAGG | *tisB* |
| ppAG72 | CCCGTTTTTATTACCTTCTTAAAGTTCTTCCCCAAAACTTTCCCCGATCCGTCGACCTGCAGTTC | Gifsy-3 |
| ppAG73 | GAGGGAGTTTTGATAAAGTTTTGATAACCGTTCGAATACTAATAATGTGTAGGCTGGAGCTGCTT | Gifsy-3 |
| pp587 | GCCTGCTTGCCGAATATC | Gifsy-3 |
| pp549 | CACGCCCGACATTATAAG | Gifsy-3 |
| pp599 | TTGGCGGTATCGGTATTG | Gifsy-3 |
| ppR25 | CGGTTCGCTTGCTGTCCATA | Gifsy-3 |
| LinkScarlet | GCGAGCATGAATTCGCGAGCATGACCGGCGGCCAGCAGATGGGCCGCATTCGCATTCATATGGTGAGCAAGGGCGAGGCA | pSeb5 |
| mScareI-HindIII-Rev | gcggtagcaagctttgctcgttacttgtacagctcgtccatgc | pSeb5 |
| tisABinterF | CTGGCGGGATTGCCCTCGGGGCGATTTTACTCCGTCTGTCATGATCGCCCTGTGTAGGCTGGAGCTGCTTCGA | *tisAB complementation* |
| tisABinterR | AACAAAAGCGGCAACAAATGTTGCCGAGAATGTGTTACGTCTGCGGCTGTCATATGAATATCCTCCTTAG | *tisAB complementation* |
| Gifsy-1 F | TAATACCGCAATACCGTTCACTACCTG | STM2605 |
| Gifsy-1 R | GTACTGTTGTCTCAGAGAATGTC | STM2605 |
| Gifsy-2 F | GTACTGTTGTCTCAGAGAATGTC | STM1048 |
| Gifsy-2 R | GTGATATCAAATTCTGTGTTCCAGATCC | STM1048 |
| Gifs-3_forward | TCTTAACTGAGCACGATATTCACCGCACC | sspH1 |
| Gifs-3_reverse | CTGACTGAAGAAGTCTCCCCTGTTTCACC | sspH1 |
| 41A | TTATCTATCTGCGCAAGGGC | ST64B (sb4) |
| 41B | CAGGTTGAGCGAGGGTTG | ST64B (sb4) |
| trpA_For | GGGAAATCTGATGGAACGCTACGAA | *trpA* |
| trpA_Rev | TTTCAGTGACTGTTCAATGCCAGGG | *trpA* |
|  |  |  |

Restriction sites are underlined.

**Reference**

1. Stanley, T.L., C.D. Ellermeier, and J.M. Slauch, *Tissue-specific gene expression identifies a gene in the lysogenic phage Gifsy-1 that affects Salmonella enterica serovar typhimurium survival in Peyer's patches.* J Bacteriol, 2000. **182**(16): p. 4406-13.

2. Ho, T.D., et al., *Identification of GtgE, a novel virulence factor encoded on the Gifsy-2 bacteriophage of Salmonella enterica serovar Typhimurium.* J Bacteriol, 2002. **184**(19): p. 5234-9.

3. Figueroa-Bossi, N. and L. Bossi, *Resuscitation of a defective prophage in Salmonella cocultures.* J Bacteriol, 2004. **186**(12): p. 4038-41.

4. Braetz, S., et al., *The role of ATP pools in persister cell formation in (fluoro)quinolone-susceptible and -resistant strains of Salmonella enterica ser. Typhimurium.* Vet Microbiol, 2017. **210**: p. 116-123.

5. Braetz, S., et al., *Prophage Gifsy-1 Induction in Salmonella enterica Serovar Typhimurium Reduces Persister Cell Formation after Ciprofloxacin Exposure.* Microbiol Spectr, 2023: p. e0187423.

6. Hautefort, I., M.J. Proenca, and J.C. Hinton, *Single-copy green fluorescent protein gene fusions allow accurate measurement of Salmonella gene expression in vitro and during infection of mammalian cells.* Appl Environ Microbiol, 2003. **69**(12): p. 7480-91.

7. Sargen, M.R. and S. Helaine, *A prophage competition element protects Salmonella from lysis.* Cell Host Microbe, 2024. **32**(12): p. 2063-2079 e8.

8. Datsenko, K.A. and B.L. Wanner, *One-step inactivation of chromosomal genes in Escherichia coli K-12 using PCR products.* Proc Natl Acad Sci U S A, 2000. **97**(12): p. 6640-5.

9. Datta, S., N. Costantino, and D.L. Court, *A set of recombineering plasmids for gram-negative bacteria.* Gene, 2006. **379**: p. 109-15.

**Supplementary Figure Legends**

**Fig S1**. **Survival upon drug treatment correlates with the ATP level and membrane potential.**

A) *S*. Typhmurium (ATCC 14028) was incubated to the indicated cell density before treatment with four-fold the MIC of ciprofloxacin (1 µg/ml). B) In parallel, the relative wild type ATP level was determined, in which the luminescence signal correlates with the ATP level. The generated luminescence signal was normalized on the number of bacteria. C) Determination of the membrane potential at different optical densities using DiOC2. To artificially reduce the membrane potential, the bacteria were pre-treated with 15 µM CCCP. At least three independent experiments were performed for each assay.

**Fig S2**. **Persister assays with D*tisB* and the complemented *tisAB* mutant.** A) The bacteria (wild type = 8640, D*tisAB* = 10752, D*tisB* = SB493) were incubated to mid-log phase and treated with 1 µg/ml ciprofloxacin. B) Treatment as in A), but with the chromosomally complemented *tisAB* strain (8640 tisAB+ = SB494, D*tisAB* *tisAB*+ = SB499). At least three independent experiments were performed for each assay

**Fig S3. ATP determination of Δ*tisB*, the complemented *tisAB* strain, and the prophage-free variants, as well as the induction of *tisB*.** A) The bacteria (cntrl = SB494, D*tisAB* *tisAB*+ = SB499, D*tisB* = SB493) were incubated to mid-log phase and treated with 1 µg/ml ciprofloxacin, indicated in the figure as + CIP. B) Treatment as in A), but with the prophage-free strains. The results were normalized to the wild type (in A) to SB494 or in B) to 11126). C) Transcriptional upregulation of *tisB* following ciprofloxacin treatment in the wild type (8640) and the respective prophage-free variant (11126). Data are presented as means ± standard deviation from at least three independent experiments. Significance was calculated with an unpaired Student's t-test.

**Fig S4.** **Persister assays and membrane potential determination.**

A) Persister assays of the *S*. Typhimurium wild type (8640) either with or without resident prophages (11126). Both strains were incubated to the stationary phase (overnight) and subsequently exposed to four-fold the MIC of ciprofloxacin. B) Determination of the membrane potential of the wild type (grey bars) and the *atp* operon mutant (9200, red bars) during exponential growth. The percentage of fluorescence positive bacteria is illustrated as relative fluorescence unit (RFU). CCCP was used to artificially reduce the membrane potential as a control. Significant differences were calculated using the unpaired student`s t-test (two tailed). Three independent experiments were conducted.

**Fig S5**. **GFP translation during and after ciprofloxacin treatment.** Bacterial strains SB22 (wild type *rpsM*::*gfp*) and SB536 (Δ*tisAB* *rpsM*::*gfp*) were treated with 1 µg/ml ciprofloxacin and harvested at the indicated time points for FACS analysis to measure GFP fluorescence. For the recovery phase, the bacteria were incubated in LB medium at 37°C. A total of 500,000 events were recorded per sample. Three independent experiments were conducted.

**Fig S6**. **ScanLag of *S*. Typhimurium before and after exposure to UV.**

The wild type was incubated to the mid. log. phase and subsequently exposed to UV. To determine the lag phase of the bacteria, the bacterial survivors were plated on LB plates and the growth was monitored using flatbed scanners. Afterwards, the average lag phase (colony appearance time) and the growth rate (growth time) were calculated. Where indicated, bacteria were pre-treated with 0.5 mM arsenate. Significant differences were calculated using the unpaired student`s t-test (two tailed). At least three independent experiments were performed.

**Fig S7. CellProfiler analysis scheme.**

**S1 Table. Strains used in this study.**

**S2 Table. Plasmids used in this study.**

**S3 Table. Primer used in this study (mutagenesis, cloning and real-time PCR).**
